# Epigenetic features are significantly associated with alternative splicing

**DOI:** 10.1186/1471-2164-13-123

**Published:** 2012-03-29

**Authors:** Yuanpeng Zhou, Yulan Lu, Weidong Tian

**Affiliations:** 1 State Key Laboratory of Genetic Engineering, Institute of Biostatistics, School of Life Science, Fudan University, 220 Handan Rd, Shanghai 2004333, China

**Keywords:** Alternative splicing, Epigenetics, DNA methylation, Nucleosome occupancy, Histone modifications, Transcription factors

## Abstract

**Background:**

While alternative splicing (AS) contributes greatly to protein diversities, the relationship between various types of AS and epigenetic factors remains largely unknown.

**Results:**

In this study, we discover that a number of epigenetic features, including DNA methylation, nucleosome occupancy, specific histone modifications and protein features, are strongly associated with AS. To further enhance our understanding of the association between these features and AS, we cluster our investigated features based on their association patterns with each AS type into four groups, with H3K36me3, EGR1, GABP, SRF, SIN3A and RNA Pol II grouped together and showing strongest association with AS. In addition, we find that the AS types can be classified into two general classes, namely the exon skipping related process (ESRP), and the alternative splice site selection process (ASSP), based on their association levels with the epigenetic features.

**Conclusion:**

Our analysis thus suggests that epigenetic features are likely to play important roles in regulating AS.

## Background

Alterations in splice site choice, referred to alternative splicing (AS), can result in mRNA is forms coding for proteins with different chemical and biological activity [1-3]. AS events can be generally classified into the following categories: cassette exon (CE), exon skipping (ES), mutually exclusive exon (ME), alternative 5' splice site selection (A5SS), alternative 3' splice site selection (A3SS) and intron retention (IR) [1,2]. Early estimates suggest that at least 60% of human genes may undergo AS [4]. Now, with the application of next generation sequencing (NGS) technologies, it has been revealed that almost all genes (95%) in the human genome will undergo AS [5], highlighting its important roles in the complexity of eukaryotic gene expression. However, although much research work has been dedicated to the understanding of the AS phenomenon, the regulatory mechanism of AS is still not fully understood.

It has been widely accepted that chromatin state plays essential roles in regulating gene expression. DNA methylation, nucleosome occupancy and modifications of histone are involved in determining the chromatin state [6,7], while some transcription factors (TFs) can bind to specific regulatory regions to interact with chromatin and regulate gene expression [8]. All these factors can be considered as epigenetic features that regulate gene expression from a broad perspective [9]. Though epigenetic signatures are mainly found to be enriched in promoters, it has become increasingly clear that they are also present in exon regions, indicating a potential link of epigenetic regulation to splicing [10-12]. For example, DNA methylation level shows distinctive differential patterns between intron and exon [13]. Histone modifications such as H3K36me3, H3K79me1, H2BK5me1, H3K27me1, H3K27me2, and H3K27me3 were found to be related with exon expression [14].

Since epigenetic features have been implied to have a connection with splicing, naturally we wonder whether they may also be involved in AS. Indeed, nucleosome occupancy level was found to be lower in cassette exons than in constitutively spliced exons [14-17]. H3K36me3 and H3K9ac were found to be related to the exon skipping event of *NCAM *[18]. The level of H3K36me3 was found to be different in mutually exclusive exons of *FGFR2*, *TPM1, TPM2*, and *PKM2 *between PNT2 and hMSC cell types. H3K36me3 was also found to be depleted in skipped exon in a genome-wide study across different species [17,19], though this finding was still in an argument [15]. Other histone methylations, such as H3K4me1, H3K4me3, H3K27me3 and H3K9me1, were found to be associated with the AS events of *FGFR2 *[20], while H3K4me3 was suggested to affect the AS events of *CHD1 *[21], and H3K9me3 was found to be associated with the multiple exon skipping of *CD44 *[22]. A recent ChIP-chip assay also found that the levels of 13 histone modifications are different in between CE and constitutively spliced exon (CNE) [23]. Despite the accumulating evidence suggesting a potential connection between epigenetics and AS, to date there have been no systematic studies on investigating the association of epigenetic features with different types of AS.

In this study, we have collected epigenetics data from six cell lines and carried out a comprehensive computational survey to investigate the association of different types of AS with epigenetic features, such as DNA methylation, nucleosome occupancy, histone modifications (including 10 histone methylations and 13 histone acetylations), and protein features (TFs) (including 9 TFs, CTCF and RNA Pol II). We find that a number of epigenetic features are strongly associated with AS. Based on their association pattern with AS events, these epigenetic features are grouped into four clusters, with the first cluster including H3K36me3, RNA Pol II, and a set of TFs and showing strongest association with all types of AS events. In addition, based on their association with the epigenetic features, the AS events are classified into two general classes: the exon skipping related process (ESRP) including ES and ME and the alternative splice site selection process (ASSP) including A3SS, A5SS and IR. Thus, our study highlights the potentially important roles of epigenetic regulation on AS, and provides list of epigenetic features strongly associated with AS, making it possible to design experiments to further investigate the molecular mechanisms of AS.

## Results

In this study, we explore whether epigenetic regulation is likely involved in AS by investigating the association of epigenetic features with AS. The epigenetic features include DNA methylation, nucleosome occupancy, histone modifications and some protein features. We focus on comparing the association level of each feature around the splice sites of between the alternatively spliced exon (ASE) and the constitutively spliced exon (CNE). Here, the types of ASE being investigated include only the alternative spliced internal exons, such as ME, ES, A3SS, A5SS and IR. ES only refers to the single exon skipping event. Details on the definition of internal exons and how to determine these ASE types can be found in **Methods **and Additional file 1. When evaluating the significance of the association, we divide the regions surrounding the acceptor and donor splice sites into the intronic and exonic regions each with a length of 100 bp (base pair), respectively, and compute a P-value in each region to indicate the significance of difference in between a type of ASE and CNE for a given feature. The difference is considered statistically significant if the P-value is smaller than 1e-2. In the following sections, unless otherwise specified, if a feature is said to be higher or lower in a given type of ASE, then it always refers to the results of comparison with the level of the feature in CNE; if no specific region, e.g., the exonic or intronic region, is mentioned, then it implies that the association is true for all the regions; in addition, if there are available data for a given feature in two or more cell lines, the results are always averaged across all available cell lines.

### The association of DNA methylation and nucleosome occupancy with AS

Here, we investigate whether the level of genomic CpG dinucleotides (termed CG) and the percentage of methylated CpG dinucleotides (termed mCG) are different in between ASE and CNE. The mCG data is obtained from a study of DNA bisulfite sequencing (BS-seq) of H1 hESC and IMR90 cells [11]. The nucleosome occupancy dataset is obtained from a study using high-throughput sequencing after MNase digestion in resting CD4+ cells [24].

**Figure 1:**
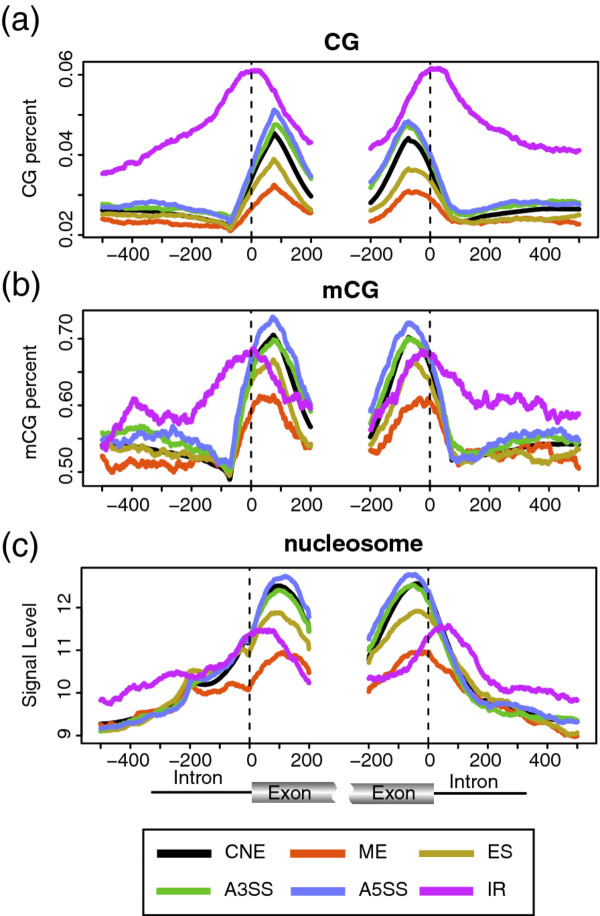
**The association of DNA methylation and nucleosome occupancy with AS**. **(a) **The distribution of genomic CpG dinucleotides level (CG) around the splice sites of different types of AS events. **(b) **The distribution of DNA methylation level (mCG) around the splice sites of different types of AS events. In both **a **and **b**, a sliding window of 147 bp is used for generating the profile for the figure; the x-axis is the position relative to acceptor site (left) and donor site (right); the y-axis is the CG percentage for **a **and the methylation percentage for **b**. (**c**) The distribution of nucleosome occupancy around the splice sites of different types of AS events. In **c**, no sliding window is used (see **Methods **for details); the x-axis is as in **a**, and the y-axis represents the ChIP signal level. (ES (exon skipping), ME (mutually exclusive exon, A5SS (alternative 5' splice site selection) A3SS (alternative 3' splice site selection), IR (intron retention)).

We find that ME and ES have significantly lower level of both CG and mCG in the exonic regions (Figure 1a-b). In contrast, IR has significantly higher level of CG in both the exonic and intronic regions; yet, its mCG level in the exonic region is significantly lower while in the intronic region is significantly higher. For both A3SS and A5SS, their associations with CG and mCG are generally not as significant as that with the other types of ASE, though the association is significant in some regions (See Additional file 2 for details). The association patterns of mCG with the ASE in individual cell lines are similar to the above results (Additional file 3).

For nucleosome occupancy, we find that its level is significantly lower in the exonic regions of both ME and ES, with the results of ME more significant than that of ES (Figure 1c). This finding is consistent with several early reports that ES has lower nucleosome occupancy [14-16]. The nucleosome occupancy level is also significantly lower in the intronic region of the donor splice site of IR. Both A5SS and A3SS do not have strong association with nucleosome occupancy. The overall association patterns of nucleosome occupancy with ASE are similar to that of mCG's, indicating a potential connection between them in AS.

### The association of histone modifications with AS

We obtain histone modification data from several recently published ChIP-seq studies [10,25,26]. We select 10 histone methylations and 13 histone acetylations that have available data in at least two cell lines (see Additional file 4 for details about the histone modifications and the corresponding cell lines). The distribution of each histone modification is drawn by averaging the ChIP signal across all cell lines.

**Figure 2:**
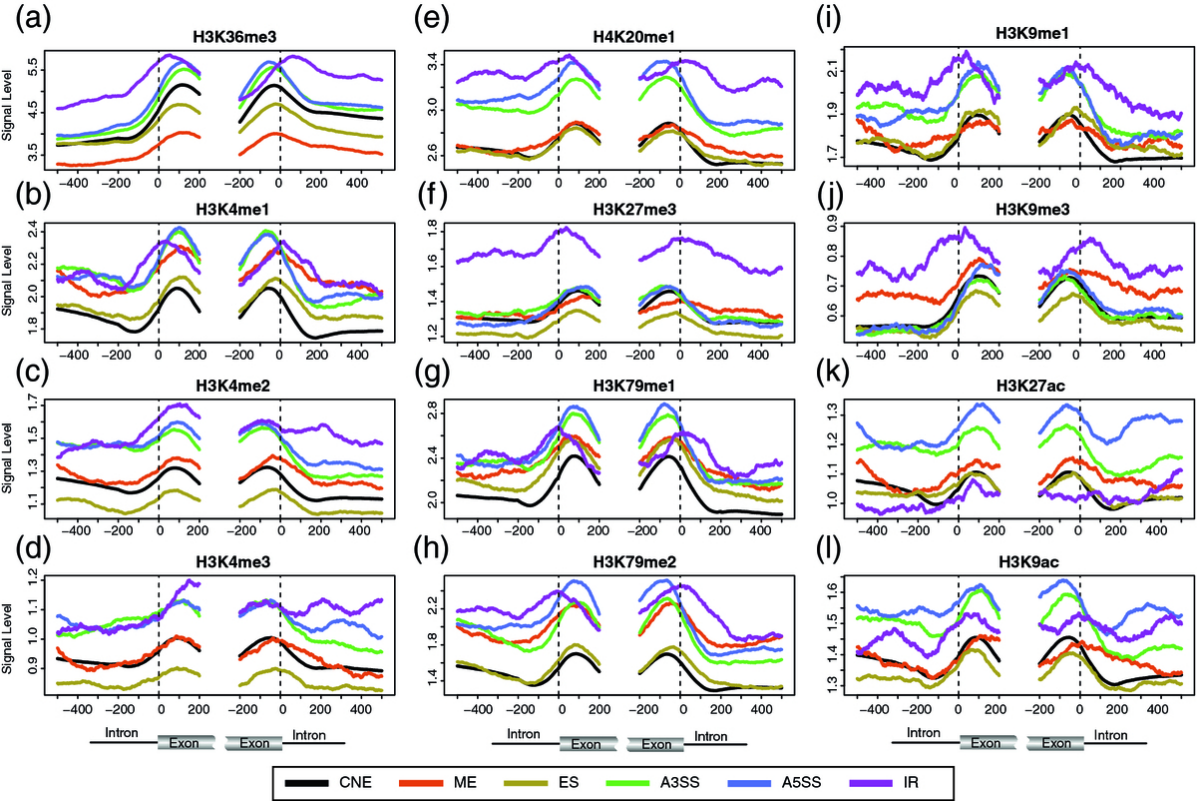
**The association of histone modification with AS**. The color scheme and plotting method are the same as that of nucleosome occupancy (Figure 1c). (ES (exon skipping), ME (mutually exclusive exon), A5SS (alternative 5' spice site selection), A3SS (alternative 3' splice site selection), IR (intron retention)).

Among the histone methylations we investigate, H3K36me3 (Figure 2a) is the only one that is significantly associated with all types of ASE in all regions, though the association patterns are different: its level is significantly lower in ME and ES and significantly higher in A3SS, A5SS and IR. A previous study also reported that ES has a lower H3K36me3 level [19]. The level of H3K4 methylations, including H3K4me1, H3K4me2, and H3K4me3 (Figure 2b-d), is almost all significantly higher in A3SS and A5SS except that the significance of the association of H3K4me3 with ASE is in between 0.01 and 0.05 in some regions. However, these H3K4 methylations show different association patterns with IR, ES and ME: the level of H3K4me1 is significantly higher in the intronic region of IR and in most regions of ME; the level of H3K4me2 is higher in the intronic region of the donor splice site of IR and significantly lower in most regions of ES; the level of H3K4me3 is significantly lower in ES. For the other histone methylations, the level of H4K20me1 (Figure 2e) is significantly higher in A3SS, A5SS and IR; the level of H3K27me3 (Figure 2f) is significantly higher in the exonic region of ES; the level of H3K79me1 (Figure 2g) is significantly higher in A3SS and A5SS, and slightly higher in the intronic region of ES; the level of H3K79me2 (Figure 2h) is significantly higher in ME, A3SS and A5SS, and most region of IR; the level of H3K9me1 (Figure 2i) is significantly higher in A3SS, A5SS and most regions of IR. However, H3K9me3 (Figure 2j) is not significantly associated with any type of ASE.

Histone acetylations are not as significantly associated as histone methylations with AS (Additional file 2). We only find that the level of H3K9ac and H3K27ac (Figure 2k-l) are higher in A3SS and A5SS, while the level of H3K14ac is slightly higher in the intronic region of ME (P = 0.0438, Additional file 5).

### The association of protein features with AS

Besides the above-mentioned well known epigenetic features, other features, such as TFs, CTCF and RNA Pol II are also thought to be related with the modification of chromatin structures [27-30]. Despite that there have been no report on the association of these TFs with the splicing machinery, given their relationships with chromatin structure, it is reasonable to hypothesize that some of them may be involved in splicing either directly or indirectly, and therefore may likely have an impact with AS. For example, a recent study reported that the rate of RNA Pol II elongation might have an impact on AS [31], while CTCF was found in a recent study to be related to the alternative splicing of *CD45 *gene [32]. However, to date the relationships of these TFs with AS have not been systematically investigated. Here, we analyze the association pattern of 9 TFs, CTCF and RNA Pol II that have ChIP-seq data [10,25,26] of more than 2 cells with AS.

**Figure 3:**
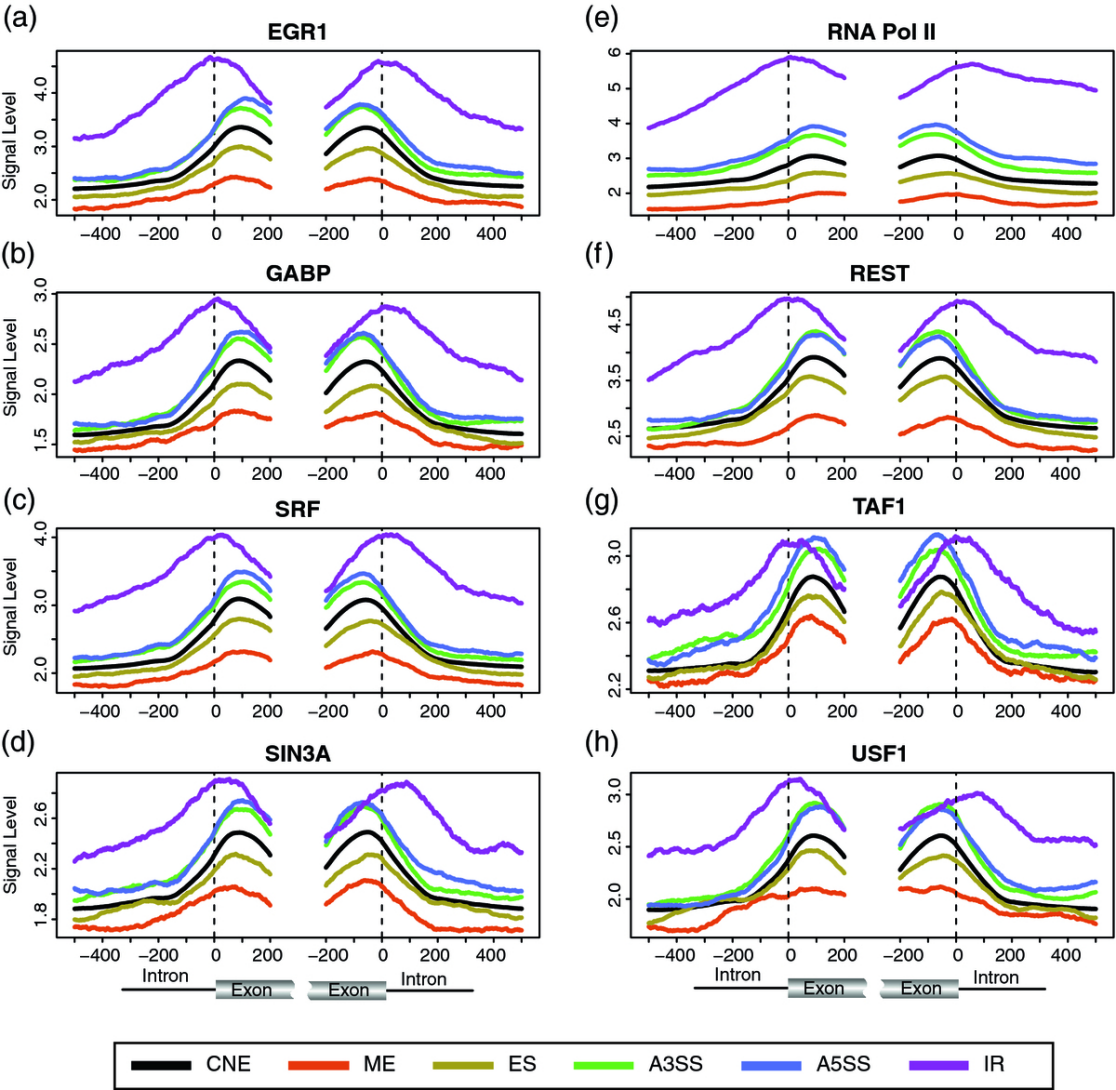
**The association of protein features with AS**. The color scheme and plotting method is the same as that of nucleosome occupancy (Figure 1c). (ES (exon skipping), ME (mutally exclusive exon), A5SS (alternative 5' splice site selection), A3SS (alternative 3' splice site selection), IR (intron retention)).

Interestingly, we find the level of EGR1, GABP, SIN3A, SRF and RNA Pol II (Figure 3a-e) are all significantly higher in A3SS, A5SS and IR, and significantly lower in ME and ES, except for SIN3A in the intronic region of the acceptor splice site of ES (P-value **= **2.54e-2); in addition, their levels all steadily increase in from ME, ES, CNE, A3SS, A5SS to IR, which is similar to the results of H3K36me3. Such association patterns also hold true in all individual cell lines (Additional file 6). Though not as significantly associated with AS as the above-mentioned proteins, REST (Figure 3f) shows higher level in A3SS, A5SS and IR, and lower level in ME and ES, but not for all regions. TAF1 shows higher level in A5SS, the exonic region of A3SS and the intronic region of IR, but lower level in the exonic region of ME (Figure 3g). Similarly, USF1 shows higher level in A3SS, IR and most regions of A5SS, but lower level in the exonic region of ME (Figure 3h). EP300 only shows higher level in the exonic region of A3SS and A5SS. In contrast, SPI1 and CTCF appear not strongly associated with any type of ASE (Additional file 7). Our CTCF result is not consistent with Shukla et al's finding that CTCF is related to alternative splicing of *CD45 *[32]. However, CTCF was found to be closely associated with histone methylation boundaries in between the active and inactive domains [10] suggesting it is less likely to exist in gene body. We also find that in general the signal level of CTCF around the splice site is very low, indicating it rarely binds near the splice site.

### Clustering of epigenetic features by its pattern in different AS types and clustering of AS types by its epigenetic pattern

**Figure 4:**
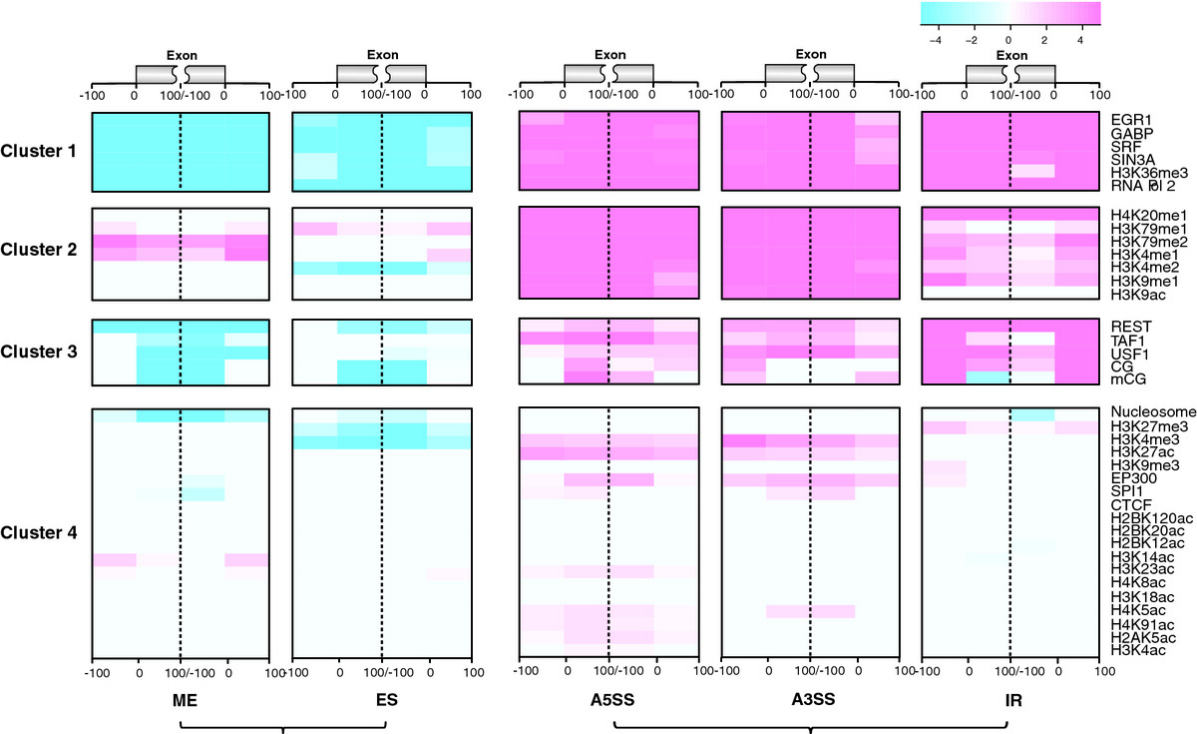
**Clustering of epigenetic features on the basis of their association with AS**. K-means clustering is used to identify four clusters of epigenetic features. The profile for clustering is generated by converting the p-value of the association of each epigenetic feature with different types of AS events in each bin around the splice sites, see **Methods **section for details. (ES (exon skipping), ME (mutually exclusive exon), A5SS (alternative 5' splice site selection), A3SS (alternative 3' splice site selection), IR (intron retention)).

By applying a k-means clustering procedure, we discover four clusters of epigenetic features on the basis of their association with ASE (Figure 4 and Additional file 8). The first cluster shows strongest association with AS, which includes four TFs (EGR1, GABP, SRF and SIN3A), RNA Pol II and H3K36me3 whose levels all show steadily increased patterns in from ME, ES, CNE, A3SS/A5SS to IR. The second cluster includes six histone methylations: H3K79me1, H3K79me2, H4K20me1, H3K9me1, H3K4me1 and H3K4me2, and one histone acetylation: H3K9ac. Features in this cluster generally show higher level in A3SS, A5SS and IR (except for H3K9ac in IR), while their association patterns with ES and ME are complex. The third cluster includes CG, mCG, REST, TAF1 and USF1, which features have similar association pattern with AS to those in the first cluster, though less significant. The fourth cluster comprises of nucleosome occupancy, EP300, SPI1, CTCF, H3K4me3, H3K9me3, H3K27me3 and 12 types of histone acetylations. Features in this cluster generally do not have strong association with AS except for nucleosome occupancy, H3K4me3, H3K27me3, H3K27ac, and EP300.

**Figure 5:**
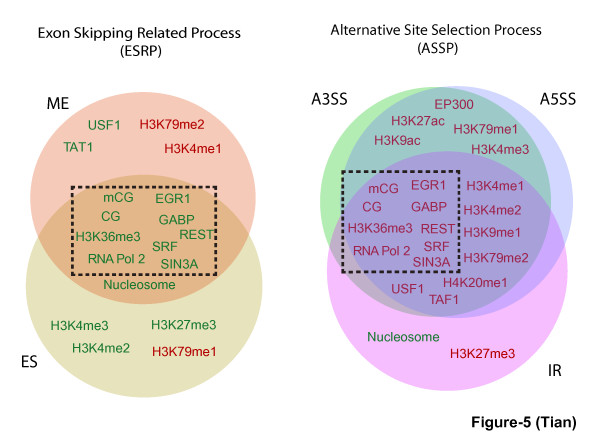
**Epigenetic features strongly associated with different types of AS**. Epigenetic features strongly associated with different types of AS events are shown inside the circle of ESRP and ASSP. The features showing higher level and lower level in AS events than in CNE are colored in red and green, respectively. The features inside the dashed black box are those common in both ESRP and ASSP; note their association patterns are very different in between ESRP and ASSP. (ES (exon skipping), ME (mutually exclusive exon), A5SS (alternative 5' splice site selection), A3SS (alternative 3' splice site selection), IR (intron retention)).

In addition to clustering epigenetic features, we cluster the AS types into two classes on the basis of their association with each epigenetic feature. One cluster includes ME and ES, while another includes A3SS, A5SS, and IR. In general ME and ES can be considered as the exon skipping related process (termed ESRP here) that involves the inclusion or skipping of one or more complete internal exons, while A3SS, A5SS and IR can be considered as the alternative splice site selection process (termed ASSP here) that involves the selection of splice site within an internal exon. Our results therefore suggest that these two classes of AS may be under different epigenetic regulatory mechanisms. In Figure 5, we summarize the epigenetic features showing unique association pattern with ESRP and ASSP, respectively. There are features, such as CG, mCG, H3K36me3, RNA Pol II and five TFs (EGR1, GABP, SRF, REST and SIN3A), that are associated with both ESRP and ASSP; however, their levels are lower in ESRP but higher in ASSP, compared to that in CNE. The epigenetic features uniquely associated with either ESRP or ASSP include nucleosome occupancy that is lower in ESRP, and five histone methylations (H4K20me1, H3K4me1, H3K4me2, H3K9me1 and H3K79me2) and two TFs (TAF1 and USF1) that are all higher in ASSP. Each type of ASE inside the same cluster also has its own uniquely associated epigenetic features. In ESRP, ME has higher level of H3K79me2 and H3K4me1; ES has significantly lower level of H3K27me3, H3K4me2, and H3K4me3, and significantly higher level of H3K79me1. Although IR is grouped together with A3SS and A5SS in ASSP based on their association patterns with epigenetic features, it also has noticeably different patterns from them. For example, IR has significantly higher level of H3K27me3, and significantly lower level of nucleosome; in contrast, both A3SS and A5SS have higher level of H3K27ac, H3K9ac, H3K79me1, H3K4me3 and EP300, suggesting that different epigenetic mechanisms may take place when regulating IR or A3SS/A5SS.

### Correcting the effect of ChIP-seq input and nucleosome occupancy

**Figure 6:**
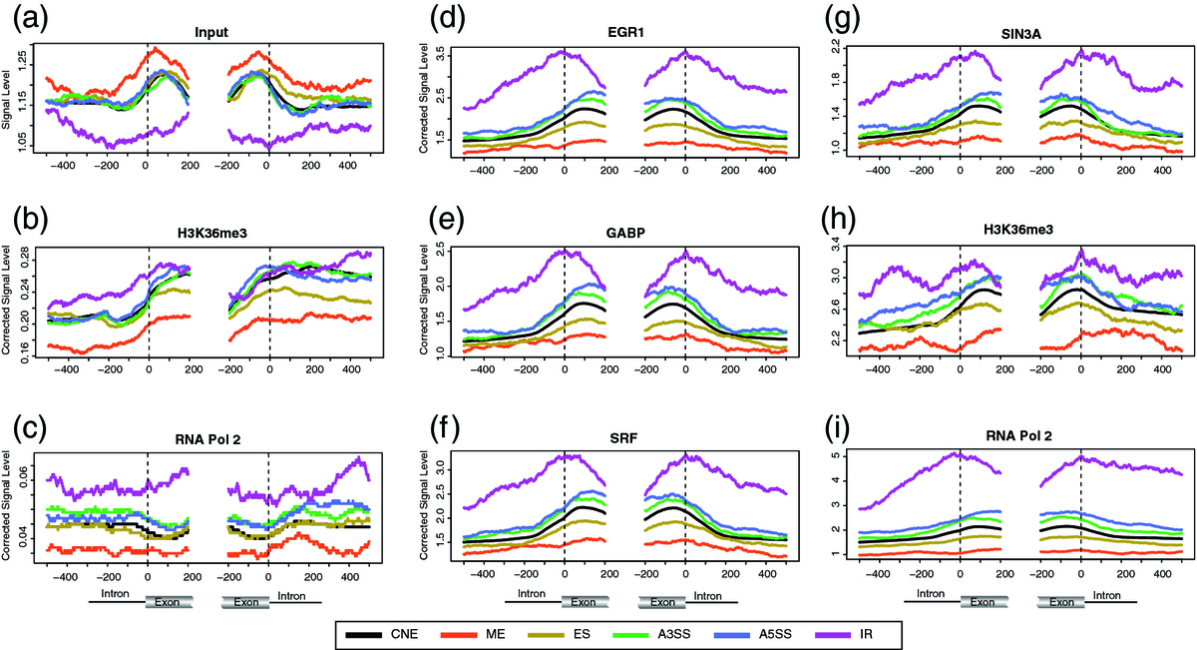
**The association of epigenetic features after correcting for ChIP-seq input and nucleosome occupancy**. (**a**) The distribution of ChIP-seq input around the splice sites of different types of AS events, the plotting method is the same as that in Figure 1c. (**b-c**) The distribution of Cluster 1 features (identified in Figure 4) corrected by nucleosome occupancy around the splice sites of different types of AS events in CD4+ cell line.*(**d-i**) The distribution of Cluster 1 features corrected by ChIP-seq input around the splice sites of different types of AS events in K562 cell line. (ES (exon skipping), ME (mutually exclusive exon), A5SS (alternative 5' splice site selection), A3SS (alternative 3' splice site selection), IR (intron retention)). * The nucleosome corrected signal level is low because the nucleosome occupancy has high signal level while the data of CD4+ are generate in 2007 and have less reads than other data source.

All above results are obtained by counting the raw sequence reads from the ChIP-seq studies of the epigenetic features. Since there may be significant background noise which may cause some bias to the results, we perform the analysis by correcting for the ChIP-seq input data. Five of six cell lines we investigate in this study have the available input data of ChIP-seq. We fist inspect whether there is any difference for the input data in between different types of ASEs and CNEs. The results show that in general, except for ME and IR, the input data have very similar distribution pattern in all ASEs and CNEs (no statistical significant difference detected, Figure 6a and Additional file 9). Interestingly, the level of input data is higher in ME than in CNE, while it is lower in IR than in CNE, which are opposite to the association patterns of most epigenetic features using the raw sequence reads. Based on the distribution pattern of the ChIP-seq input data across different types of ASEs and CNEs, our major findings should remain the same. To further illustrate the effects of input data, we select two cell lines, GM12878 and K562, that have available data of the epigenetic features belonging to Cluster 1 (the cluster with the most significant association with AS) and the input data, and perform the analysis again by correcting for the input data. The distribution patterns of these epigenetic features around the splice site after correcting for the input data remain the same as before (Figure 6d-i, Additional file 10). The statistic analysis also shows that before and after the correction, the p-values of these epigenetic features in the two cell lines are similar (Additional file 11), suggesting that our major conclusions are unlikely to be affected by correcting for the input data.

It has been reported that most histone modifications are related to the positioning of nucleosomes [14,15]. In this study, nucleosome occupancy is found to have similar association patterns with AS to that of most modifications (Figure 1c), though its significance level is considerably lower. To investigate how significant nucleosome occupancy may contribute to the association of other features with AS, we perform the analysis by correcting for nucleosome occupancy. However, since nucleosome data is available only for CD4+ cell lines, among the significantly associated epigenetic features we only do the correction for H3K36me3 and RNA Pol II which have available data in that cell line. The results show that the association patterns of these two features remain the same as before, and the statistic tests are still significant, though the significance level of ES, ME and A5SS is lower than before (Figure 6b-c). Additionally, for all other epigenetic features with available data in CD4+ cell lines, the results show that after the correction for nucleosome occupancy, their association patterns are still similar to before (Additional file 12). Thus, we conclude that nucleosome alone is not enough to explain the association of other epigenetic features with AS.

## Discussion

Prior to our work, there have been a number of studies on how epigenetic features may affect splicing [20,31,33]. It has been proposed that specific histone modifications may help to recruit related TFs or splicing factors or to change the RNA Pol II elongation efficiency, which can affect splicing. As RNA Pol II elongation efficiency may play a role in affecting splicing, we reason that higher level of RNA Pol II around the splice sites may correspond to lower RNA Pol II elongation rate, which can help promote splicing efficiency. Based on our findings that the level of RNA Pol II is significantly higher in ASSP while significantly lower in ESRP, here we propose that lower RNA Pol II elongation rate may be necessary for selecting the alternative splice site, while higher RNA Pol II elongation rate can facilitate the exon skipping events. Specific histone methylations, such as H3K36me3, may help to recruit general TFs like EGR1, GABP, SRF, SIN3A, REST, TAF1 and USF1, which can help to retain RNA Pol II around the splice site and promote ASSP; while other histone methylations, such as H4K20me1, H3K4me1, H3K4me2, H3K9me1 and H3K79me2, may provide additional help for this process. In the absence of H3K36me3, the above-mentioned general TFs may not be recruited, making it easy for RNA Pol II to pass through the splice site, and resulting in ESRP events. Our model explains the general difference between the ASSP and ESRP events.

In this study, the transcriptional effects are not taken into consideration when analyzing the association of epigenetic features with different types of ASEs. This is because of the following reasons. Firstly, not all cell types investigated in this study have the available exon-array or RNA-seq data that were obtained under the same condition as that of the epigenetic features, making it difficult to reduce the transcriptional effects across different cell types. Secondly, even in cell line in which the exon-array or RNA-seq data were available, we find that there are usually significant variations of expression level in between the individual exons of a given gene, making it impossible to reduce the transcriptional effects on different types of exons. Thirdly, transcription level is a result of both epigenetic regulation and splicing, even with available data it may be difficult to distinguish the effects of epigenetic regulation from the effects of transcription level. Nevertheless, by focusing on comparing the distribution of epigenetic features in between a specific type of AS exons and the CNEs that are determined by comparing all Ensemble transcripts between each other, we are able to identify the statistically significant differences for some epigenetic features that are consistent across different cell lines. This highlights the likely difference of epigenetic regulation on potentially alternatively spliced exons from that on constitutively spliced exons.

Exon length may be another factor that affects the results of our analysis, because we do not separate exons by using different cutoff. However, the short exons (e.g., with a length shorter than 50 nt) and the long exons (e.g., with a length longer than 300 nt) only account for a small portion of all exons (Additional file 13), and therefore are unlikely to affect the results. In our analysis, we define the regions surrounding the splice site to be ± 100 nt to the splice site. When the regions are defined to be ± 50 nt to the splice site, the results remain almost the same (Additional file 2). In addition, Kolasinska-Zwierz et al. studied the alternative splicing of exons longer than 200 nt, which excluded most exons (the typical length of an exon is 147 nt), and found that the level of H3K36me3 is lower in ES compared to CNE [19], which is consistent with our findings, further suggesting that exon length is unlikely to affect our conclusions. On the other hand, it has been shown that most epigenetic features have completely different patterns in both TSS and PolyA than in gene body [10], while the levels of some histone modifications are increased from TSS to PolyA while the levels of some are decreased, hinting that the positional effects on the level of epigenetic features may cause some bias to our conclusions. In our analysis, we exclude AS types such as alternative first exon and alternative last exon and only consider exons that are not within 2000 bp to TSS and PolyA. To further clarify the possible positional effects, we group ASEs and CNEs based on their positions (2^nd^-4^th ^exons), and repeat the analysis for the most significant epigenetic features (cluster 1 and cluster 2) on two cell lines (GM12878 and K562). The results suggest that their patterns at different positions exons remain the same as before (Additional file 14), indicating that the positional effects of the distribution of epigenetic features are unlikely to affect our conclusions. However, our analysis only focuses on the protein coding transcripts that are alternatively spliced, and the conclusion may not be directly applicable for AS of non-coding transcripts which were recently found to have different epigenetic signatures from protein coding transcripts [14,16]. On the other hand, we focus only on the regions surrounding the splice sites, and do not consider the epigenetic features in the intronic regions far from the splice sites, which may also contribute to AS.

In this study, we have uncovered a number of epigenetic features strongly associated with different types of AS. Some of the findings are consistent with other studies. For example, our finding on H3K36me3's association with ES is consistent with Kolasinska-Zwierz et al.'s [19], though their study involved less number of exons. A recent study performed by Dhami et al. found that 13 histone methylations are associated with the splicing of cassette exon [23] in which nine of them are investigated in this study. Since Dhami et al. did not report the p-value of the associations, we compare the tendency of the association of these epigenetic features in their study with ours. Except for H3K4me1, the tendency of all the other features is consistent with ours. However, because of the adjustment of multiple tests, four of them, H3K4me3, H3K27me3, H3K36me3 and H3K79me1, are significant in our study. The possible difference can be attributed to the following reasons: Dhami et al. used the ChIP-chip data, while we use the ChIP-seq data; they analyzed the AS events of only 268 well expressed genes, while we carry out a genome-wide study; they analyzed three cell lines, while we analyze six cell lines. It is worth noting that these recent studies are limited because their studies either focused on data from single cell line [19], or used ChIP-chip data which are considered to have lower quality compared to ChIP-seq data [23]. In contrast, our study uses the ChIP-seq data from six cell lines to investigate the associations of not only histone methylations but also other epigenetic features, such as TFs, with AS, and discovers a number of epigenetic features significantly associated with AS, in which many of them are first reported, including EGR1, GABP, SRF, SIN3A, etc. These discovered significant epigenetic features will be useful to guide experimentalists to design specific experiments to further explore the mechanisms of epigenetic regulation on AS.

### Gene names

*NCAM*, Neural cell adhesion molecule; *FGFR2*, Fibroblast growth factor receptor 2; *TPM1*, Tropomyosin 1 (alpha); *TPM2*, Tropomyosin 2 (beta); *PKM2*, Pyruvate kinase muscle; *CHD1*, Chromodomain helicase DNA binding protein 1; *CD44*, CD44 molecule (Indian blood group)

### Protein names

CTCF, CCCTC-binding factor (zinc finger protein); EGR1, Early growth response 1; GABP, GA binding protein transcription factor; SIN3A, SIN3 homolog A transcription regulator; SRF, Serum response factor (c-fos serum response element-binding transcription factor); REST, RE1-silencing transcription factor (NRSF); TAF1, TAF1 RNA polymerase II TATA box binding protein (TBP)-associated factor; USF1, Upstream transcription factor 1; EP300, E1A binding protein p300 (P300); SPI1, Spleen focus forming virus (SFFV) proviral integration oncogene spi1 (PU.1)

## Conclusions

Our results reveal that epigenetic features are strongly associated with AS, suggesting that epigenetic regulation may be involved in AS. By applying a clustering procedure, we identify four tight clusters of epigenetic features, with features in the first cluster showing strongest association with AS. The AS events are grouped to two classes: the exon skipping related process (ESRP) (including ME and ES) and the alternative splice site selection process (ASSP) (including A3SS, A5SS and IR) on the basis of their association patterns with epigenetic features, indicating that these two processes may involve different mechanisms of epigenetic regulation.

## Methods

### Annotation of alternative splicing (AS) events

All transcripts used to annotate genome-wide AS types are downloaded from Ensembl (build 59) annotations and only coding transcripts are selected [34]. We identify AS events, such as ME, ES, A3SS, A5SS, IR only from internal exons that are not located within the region of 0-2000 bp to TSS or -2000-0 bp to the terminal site of a transcript. Because CE is complex, and include any AS events in which an exon is either skipped or included [1], e.g., both ME and ES are subtypes of CE, it is not analyzed in this study. For comparison, we identify CNE from the internal exons as well. The definitions of ASE follow a previous study [35]. The procedures on identifying AS events and CNE involve the following steps (Additional file 1a). First, we map known transcripts to the reference genome sequences (hg19). Then, the exon-intron junction sites of the transcripts belonging to the same coding gene region are compared with each other. Next, the splicing events are first coded into binary codes and then converted to decimal code for convenience, see the Nagasaki's method [35] for details. Here, we employ a slightly modified version of the Nagasaki's method (see Additional file 1a), such that less information will get lost and complex situations can be avoided. Finally, we recognize the AS event according to the decimal code as shown in Additional file 1b. The numbers of CNE, ES, ME, A3SS, A5SS and IR we identify are 103,806, 2725, 1206, 2521, 1884 and 563, respectively. Then, we focus on the splice site of the above events for further analysis. Here, for A3SS and A5SS, we always choose the splice site corresponding to a longer exon in the analysis; for IR, the 3' acceptor corresponds to the 3' of the retained intron, while the 5' donor corresponds to the 5' of the retained intron; for ME, both exons are considered as independent counts, because it is impossible to know which exon is included and which one is not. The recognition script of AS is available online (Additional file 15).

### Genomic and epigenomic data preparation

CpG dinucleotides are directly counted from the hg19 genome downloaded from UCSC genome center [36]. The DNA methylation data of human H1 embryonic stem cell and IMR90 fetal lung fibroblasts cell are downloaded from SALK institute [11]. As previous studies have suggested that the sense and antisense CpG methylation are coupled [11,37], we consider cytosine methylation from both strands of CpG dinucleotides as one functional site. The nucleosome occupancy data of resting CD4+ cell are get from one publication [24]. The ChIP-seq data of histone modifications and protein features from six cell lines are downloaded from various sources: the data of resting CD4+ cell are from several publications [10,38]; the data of IMR90 cell are download from the NIH Roadmap Epigenomics [39]; the data of GM12878, K562, H1 hESC and Hep G2 cells are downloaded from UCSC genome center [36], which are generate by the Encode Project [25,26] (see Additional file 4 for details about the available data of histone modifications and protein features from each cell). The data of histone modifications and protein features are all raw alignments of sequencing reads generated by ChIP-seq. To avoid the loss of information, all alignments are extended to 200 bp according to the direction of strand [39]. If the ChIP-seq data are based on hg18, we use the liftOver tool downloaded from the UCSC genome browser [36] to convert the hg18 coordinates to hg19's. All processing scripts are written by Perl programming language, and can be obtained upon request.

### Statistical analysis

We divide the regions surrounding each splice site (including both donor and acceptor splice sites) into the exonic and intronic regions with 100 bp length each. Then, we map the epigenetic features investigated in this study to those regions. To obtain the level of an epigenetic feature in a given region of a splice site, for CpG dinucleotides, we directly count the number of CpG dinucleotides in the region; for methylated CpG dinucleotides, we calculate the percentage of CpG dinucleotides that are methylated in the region; for all other epigenetic features, we calculate the total number of raw sequencing reads mapped the region. Next, to test the significance of the association of an epigenetic feature with ASE, we collect the level of the feature of all splice sites belonging to the ASE and CNE from all cell lines available, respectively, and employ a one-tailed t-test with Bonferroni adjustment to examine the difference between the mean level of the ASE and of CNE. When combining the data for a given epigenetic feature, its data from each splice site in a cell line are considered as an independent observation and are put together for all cell lines. Therefore, if the pattern is consistent among all cell lines, the p-value will be more significant. Conversely, even if an epigenetic feature may be significant in one cell line, with the above-described treatment, its p-value will be less significant if the data from more cell lines are combined. The correction of ChIP-seq input is done by subtracting the signal of each feature by the signals of ChIP-seq input, because many splice sites do not have ChIP-seq signals. The correction of nucleosome occupancy is done by dividing the signal of epigenetic features to that of nucleosome level, because we only consider the histone features with the evidence of existing nucleosomes.

### Data visualization

To visualize the distribution of the level of the epigenetic features around the splice sites, all epigenetic features are first mapped to the -500-200 bp and the -200-500 bp region surrounding the acceptor and donor splice sites, respectively. Then, for a given type of ASE or CNE, at each nucleotide position, for CpG and methylated CpG dinucleotides, we compute the percentage of CpG and methylated CpG dinucleotides across all splice sites belonging to the ASE or CNE, followed by the application of sliding window of 147 bp, which is the typical length of an exon [24], to reduce noise; for all other epigenetic features, at each nucleotide position we compute the average number of raw reads overlapped with this position across all splice sites belonging to the ASE or CNE, without the use of sliding window because all raw reads have already been extended to 200 bp. When data are available for multiple cell lines for a given feature, the above signal levels are averaged across all cell lines. For the correction using both ChIP-seq input and nucleosome occupancy, the corrected plots are the result of divided raw reads to ChIP-seq input reads or nucleosome occupancy at each position. The heatmap illustrating the associations of all epigenetic features with all types of ASE is generated from a matrix of p-values obtained from the one-tailed t-test with Bonferroni adjustment. Here, the p-values are converted to -log (p-value) or log (p-value) if a feature has higher level in an ASE or in CNE, respectively. The heatmap is drawn using the "heatmap.2" function of R statistic language. K-means clustering is done by applying the "kmeans" function of R with 'center = 4'. The distance matrix used by K-means is converted from the heatmap matrix using 'dist' function of R with Euclidian distance.

## Abbreviations

AS: Alternative splicing; ASE: Alternative spliced exon; CE: Cassette exon; ES: Exon skipping; ME: Mutually exclusive exon; A5SS: Alternative 5' splice site selection; A3SS: Alternative 3' splice site selection; IR: Intron retention; ESRP: Exon skipping related process; ASSP: Alternative splice site selection process; TSS: Transcription start site.

## Competing interests

The authors declare that they have no competing interests.

## Authors' contributions

WT conceived the study. YZ designed experiments and carried out the analysis. YL participated in the analysis. YZ and WT drafted the manuscript. All authors read and approved the final manuscript.

## Supplementary Material

Additional file 1 **The procedures on recognizing the AS events**. (**a**) Junction site annotation and alternative splicing recognition process. (**b**) The recognition code of AS events and the number of each type of splicing event. Click here for file

Additional file 2 **All p-values indicating the association of epigenetic features investigated in this study with AS**. Click here for file

Additional file 3 **The association of mCG with AS in different cell types**. The distributions of mCG in H1 hESC and IMR90 cell types are showed; the color scheme and the plotting method is the same as that of mCG in Figure 1b. Click here for file

Additional file 4 **Available data of cell lines for all epigenetic feature and the used file name of the download file in ENCODE and Roadmap Epigenomics**. Click here for file

Additional file 5 **The association of additional histone acetylations with AS**. This figure shows the distributions of histone acetylations not included in Figure 2. Click here for file

Additional file 6 **The association of additional protein features with AS**. This figure shows the profiles of protein features not included in Figure 3. Click here for file

Additional file 7 **The association of histone modifications and protein features with AS in different cell types**. The level of each feature in four bins around the accepter and donor splice sites are shown for different cell lines; the error bars are drawn for 5% confidence interval. Click here for file

Additional file 8 **Classical multidimensional scaling plot of epigenetic features on the basis of their association with AS**. The color and point type of each epigenetic features is the same with the cluster index of the k-means clustering result (Figure 4). The distance of two features indicates the closeness of their relationships. Note that cluster 1 and cluster 2 features are distantly to each other while cluster 3 and cluster 4 features are more closely related. Some features, such as nucleosome occupancy and NRSF, are located in between clusters. Classical multidimensional scaling is done using "cmdscale" function in GNU R. Click here for file

Additional file 9 **The distribution of ChIP-seq input data in different types of ASEs and CNEs**. Click here for file

Additional file 10 **The distribution of ChIP-seq data surrounding the splice sites of ASEs and CNEs in GM12878 and K562 cell lines**. The patterns of most features are not changed after the correction. Only GM12878 and K562 cell lines are selected because they have available data for most of our significantly associated feature. Click here for file

Additional file 11 **Heatmap of epigenetic features corrected by ChIP-seq input**. Un-adjusted P-values are shown. The method is the same as that in Figure 4. Click here for file

Additional file 12 **Heatmap of epigenetic features corrected by nucleosome occupancy**. Un-adjusted P-values are shown. The method is the same as that in Figure 4. Click here for file

Additional file 13 **The distribution of exon length**. Click here for file

Additional file 14 **Statistic test of the difference between the fourth exon and third exon, for each ASE(CNE) and each feature**. Click here for file

Additional file 15 **Additional scripts for the recognitions of AS event**. Click here for file
