# Supplementary material for: Epigenetic features are significantly associated with alternative splicing
Source: BMC Genomics. 2012 Mar 29;13:123. doi: 10.1186/1471-2164-13-123 (PMC3362759; doi:10.1186/1471-2164-13-123)
Supplement: Additional file 1 — The procedures on recognizing the AS events. (a) Junction site annotation and alternative splicing recognition process. (b) The recognition code of AS events and the number of each type of splicing event. [file 1471-2164-13-123-S1.PDF]

(a)

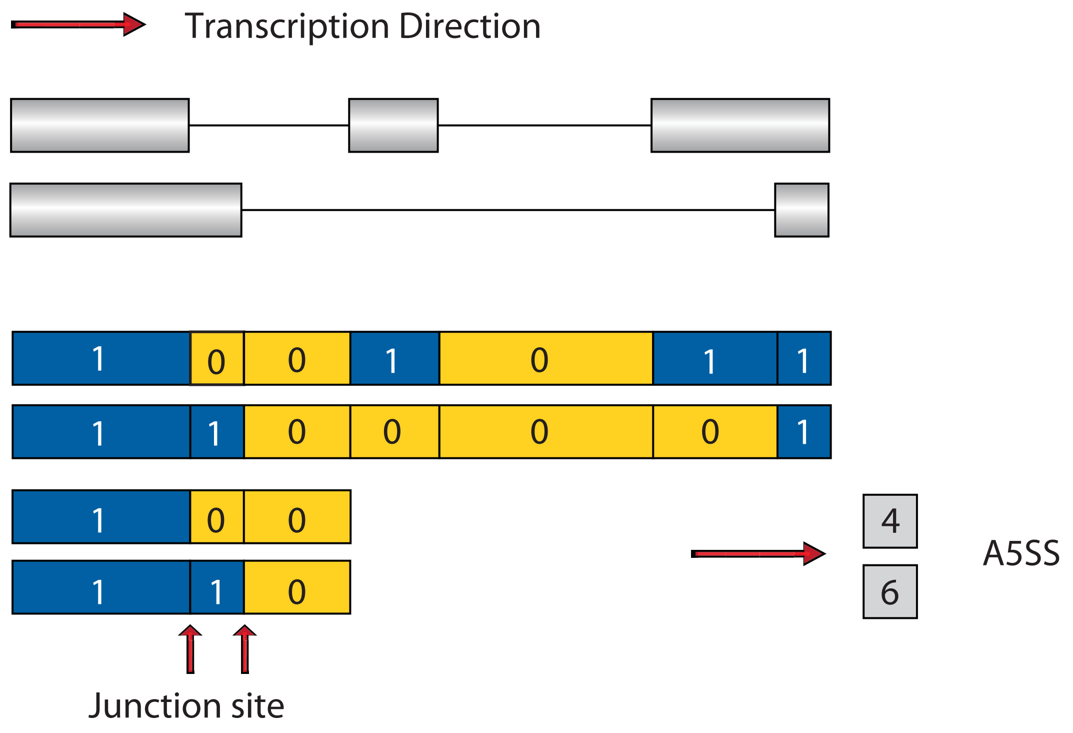


(b)

| Sketch map | Recognition Code | AS Type | Number of Events |
|---|---|---|---|
| 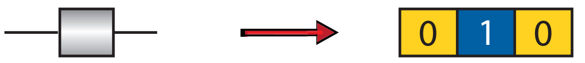 0 1 0 | 2 | CNE | 103,806 |
| 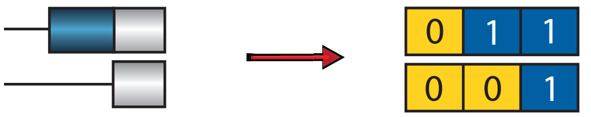 0 1 1 / 0 0 1 | 6 / 2 | A3SS | 2,521 |
| 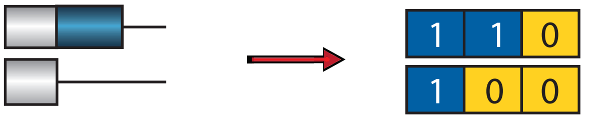 1 1 0 / 1 0 0 | 6 / 4 | A5SS | 1,884 |
| 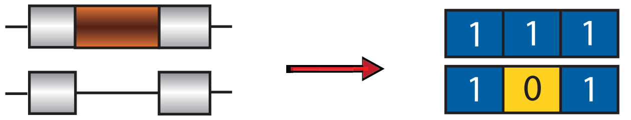 1 1 1 / 1 0 1 | 7 / 5 | IR | 563 |
| 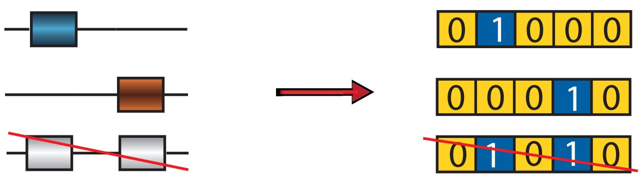 0 1 0 0 0 / 0 0 0 1 0 / 0 1 0 1 0 | 8 / 2 / 10 | ME | 1,206 |
| 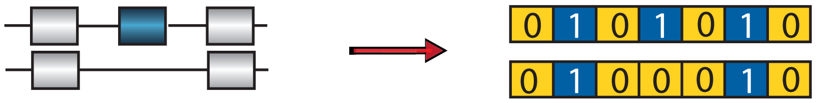 0 1 0 1 0 1 0 / 0 1 0 0 0 1 0 | 42 / 34 | ES | 2,725 |

**Supplementary Figure-1 (Tian)**
