# Supplementary figures and images for: Epigenetic features are significantly associated with alternative splicing
Source: BMC Genomics. 2012 Mar 29;13:123. doi: 10.1186/1471-2164-13-123 (PMC3362759; doi:10.1186/1471-2164-13-123)

# mCG\_H1 hESC

(a)

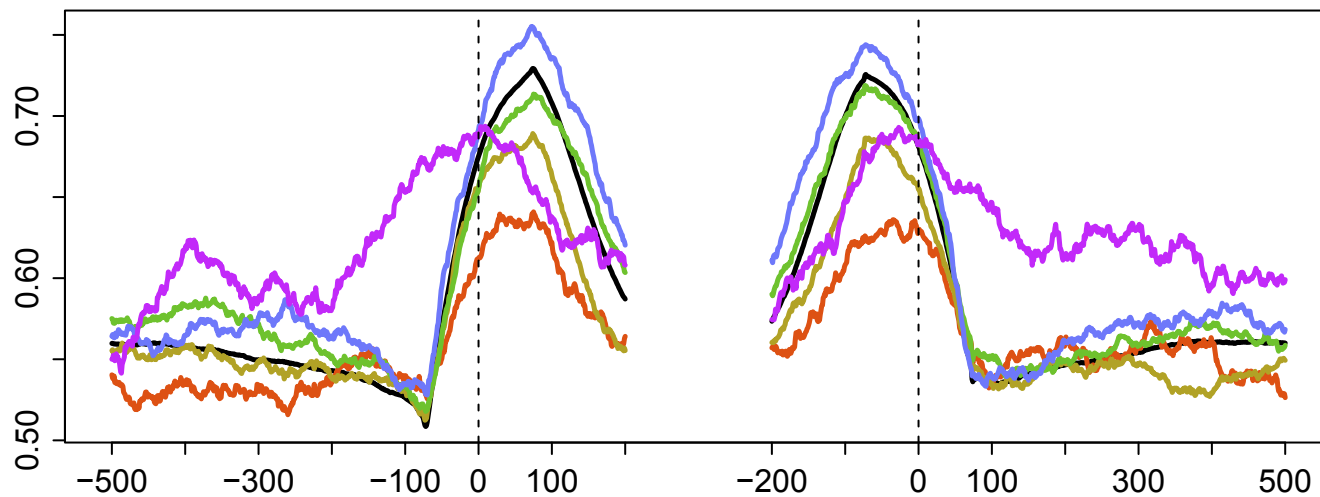

# mCG\_IMR90

(b)

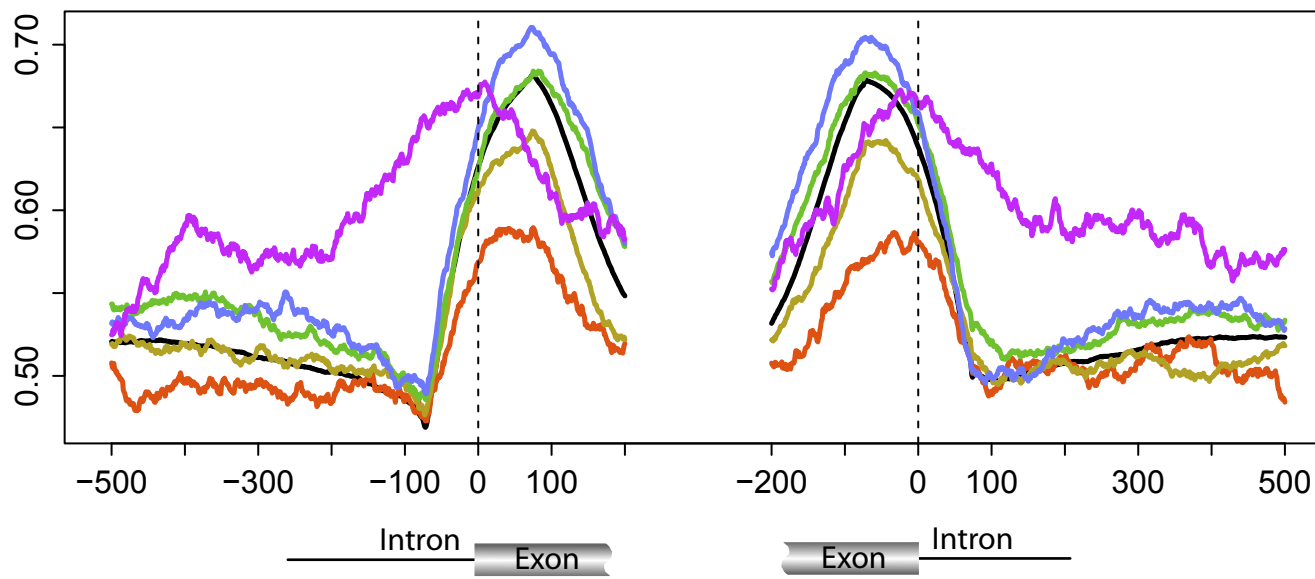

— CNE — ME — ES — A3SS — A5SS — IR

Supplement: Additional file 3 — The association of mCG with AS in different cell types. The distributions of mCG in H1 hESC and IMR90 cell types are showed; the color scheme and the plotting method is the same as that of mCG in Figure 1b. [file 1471-2164-13-123-S3.PDF]

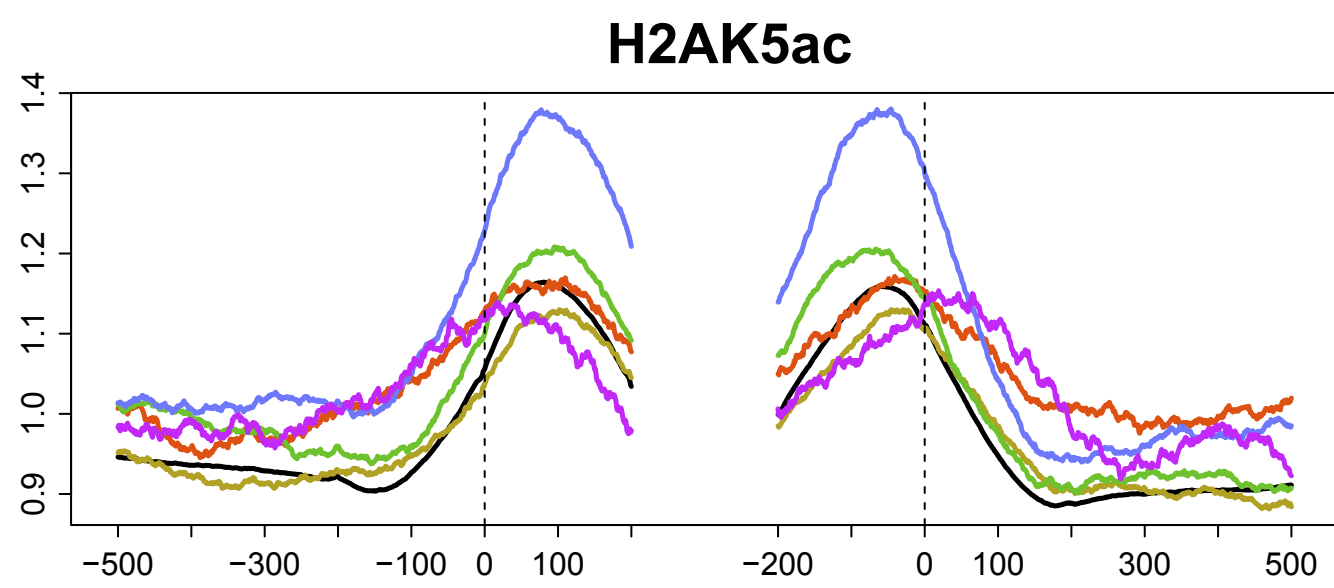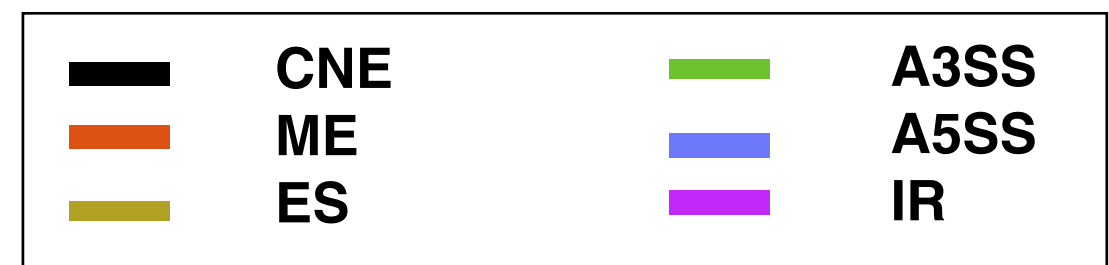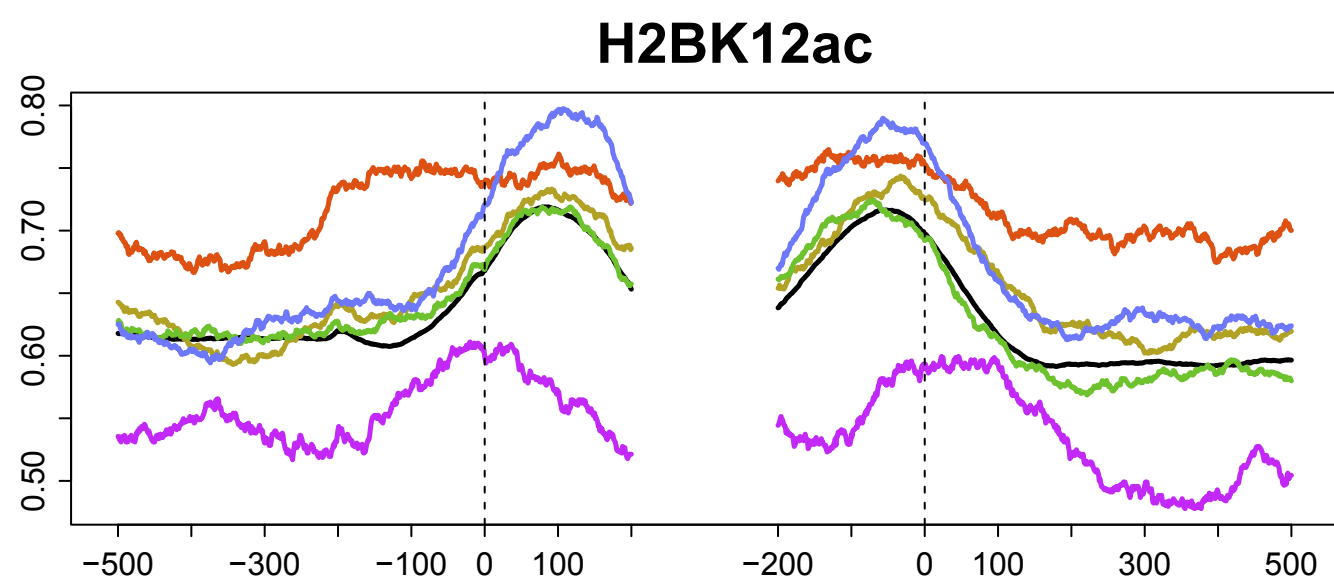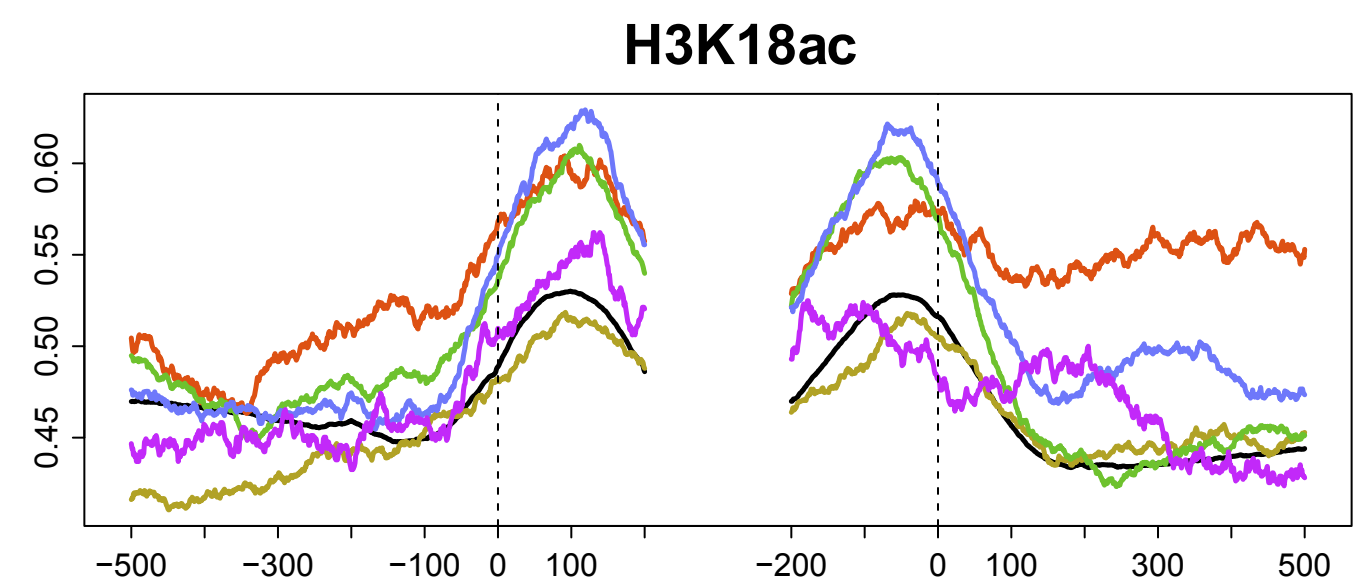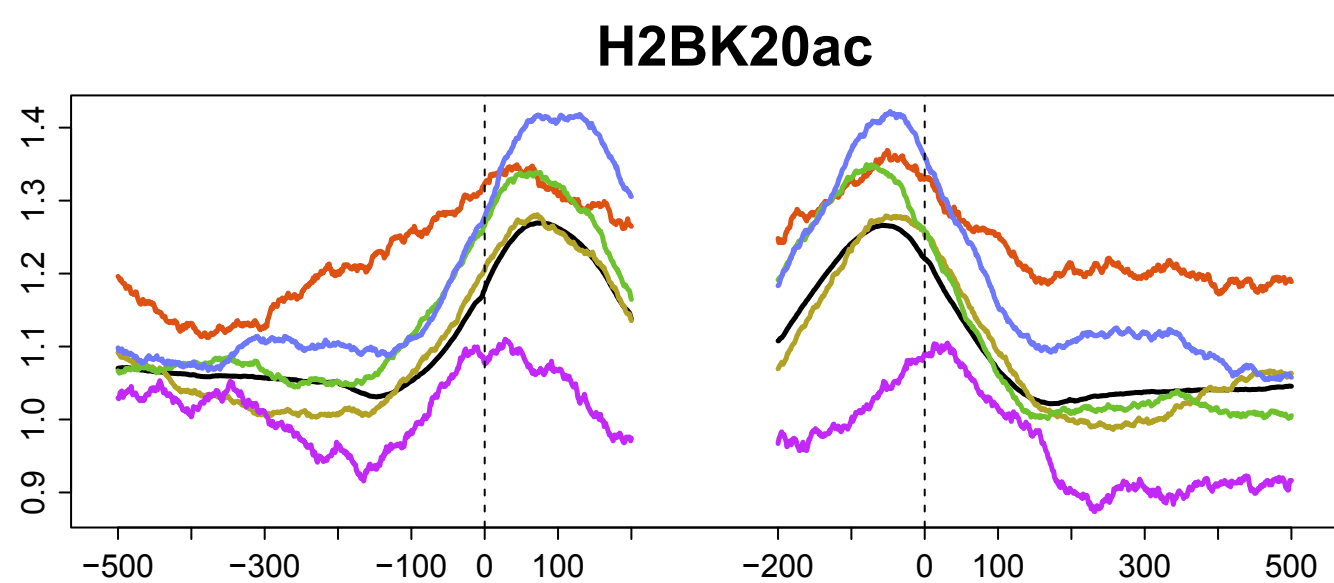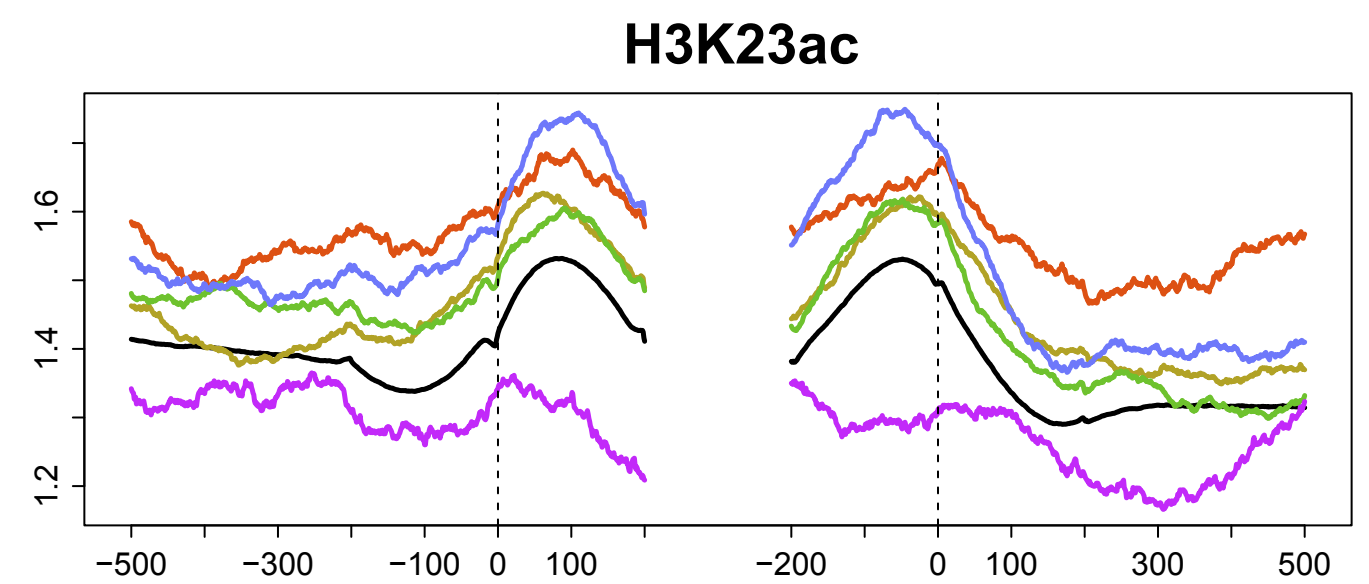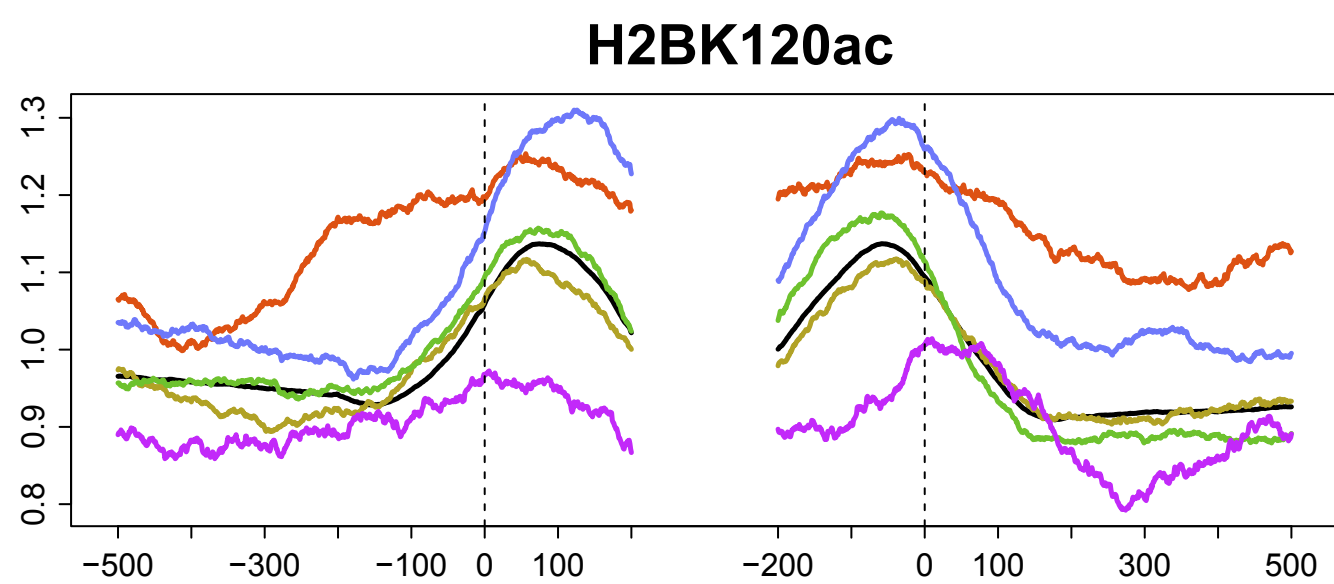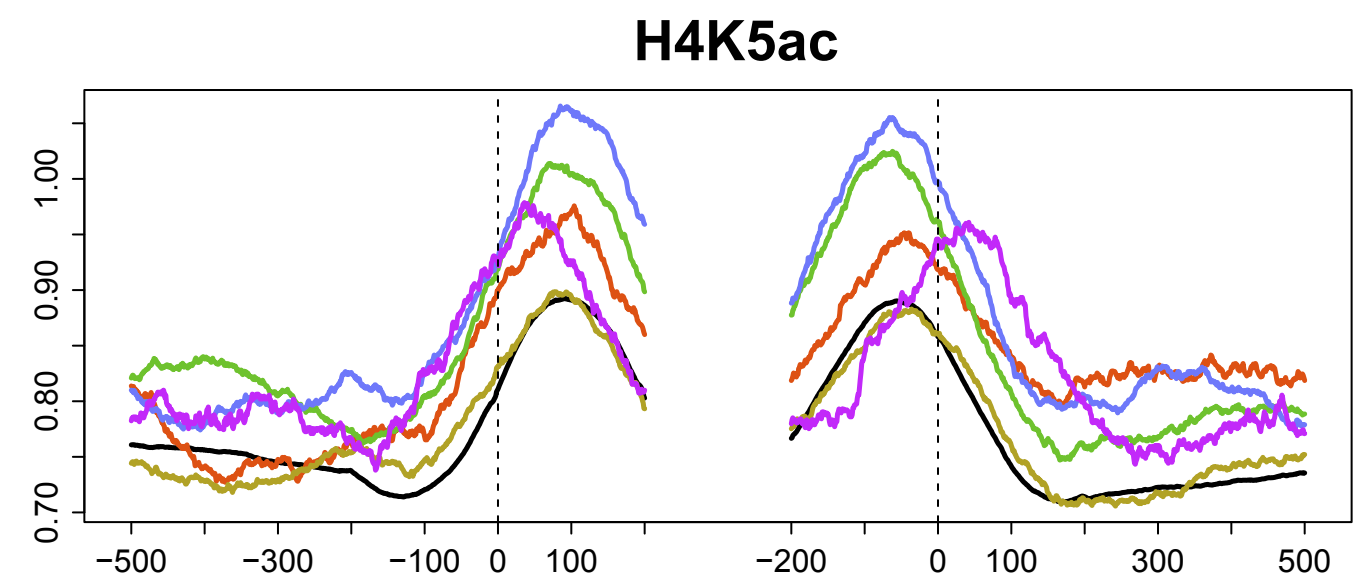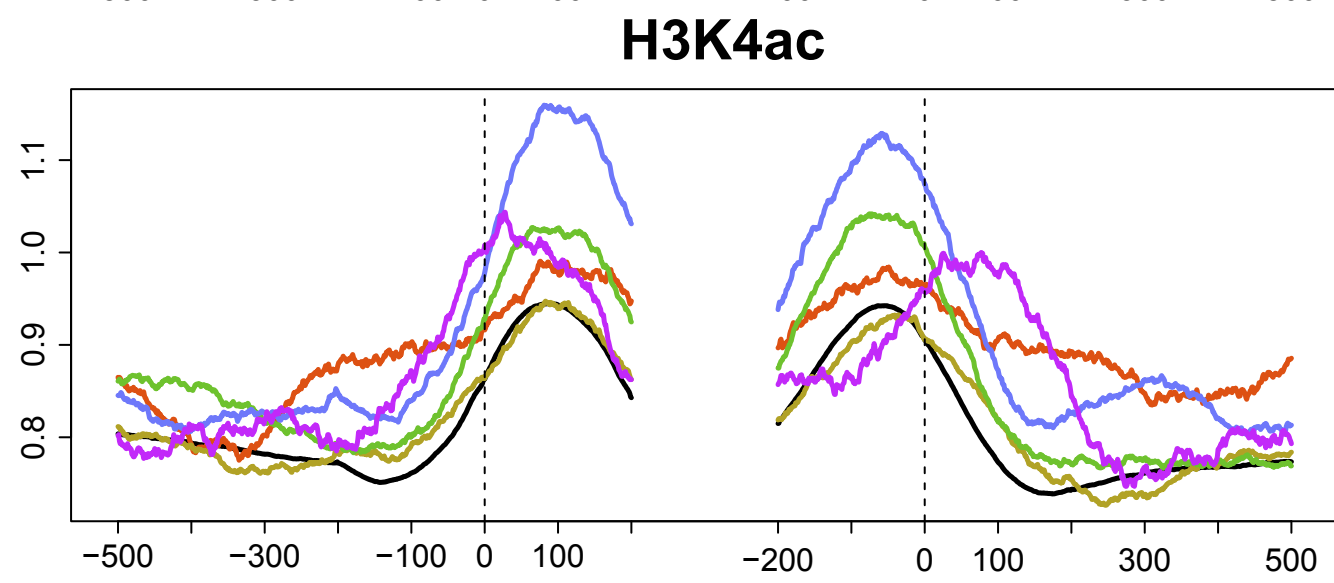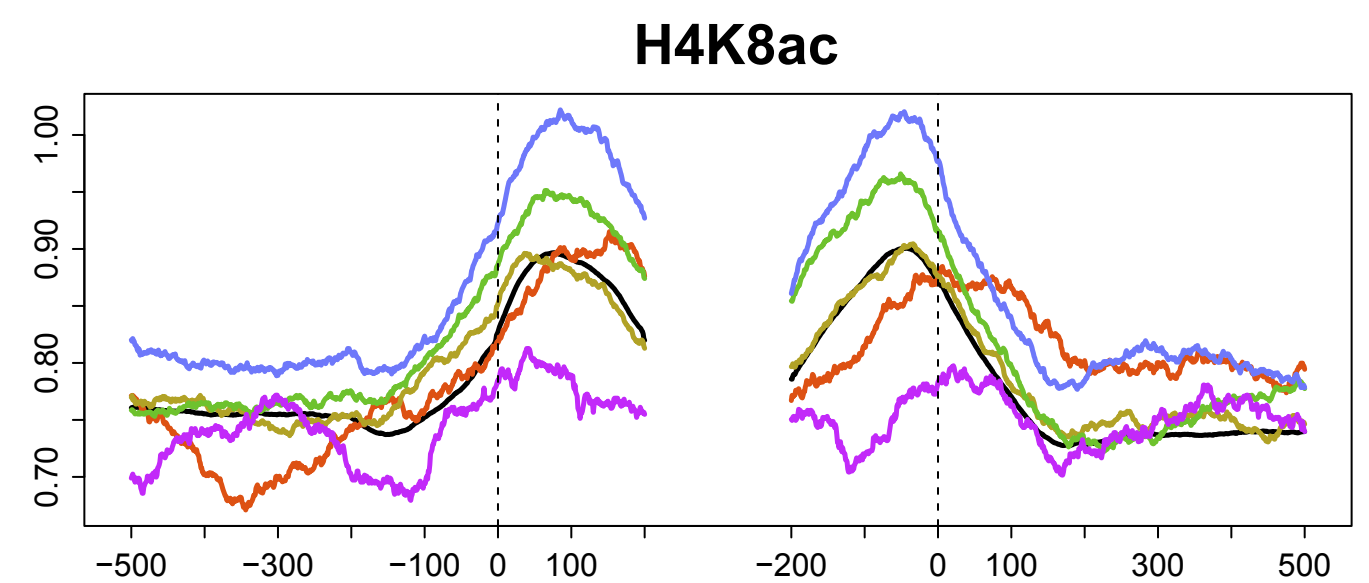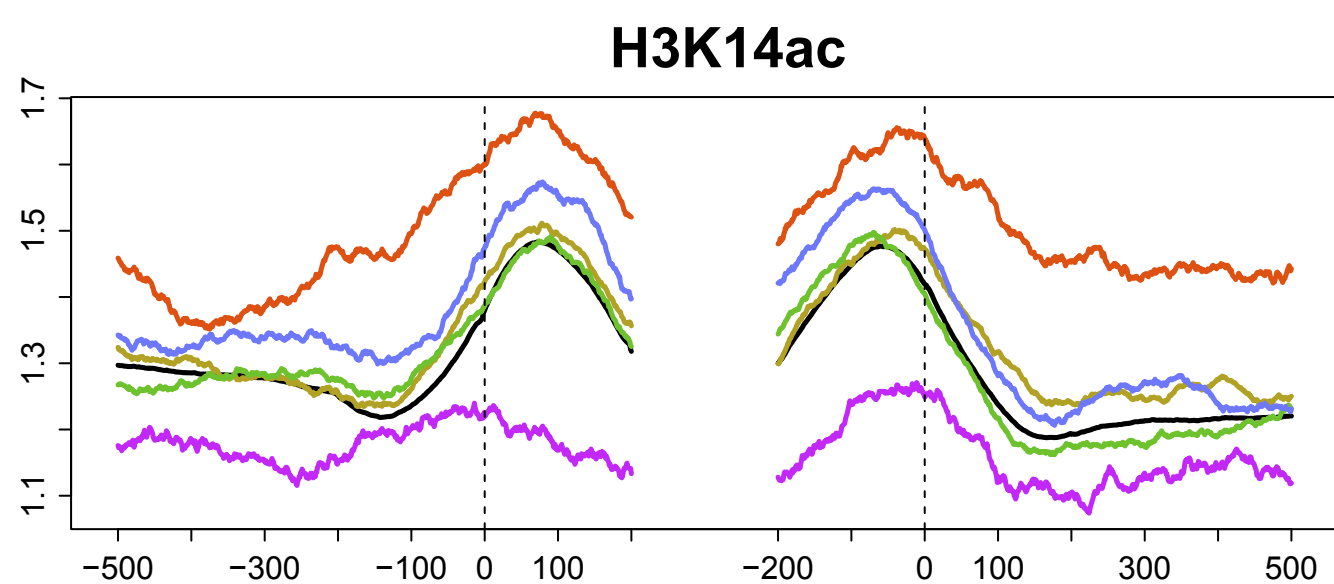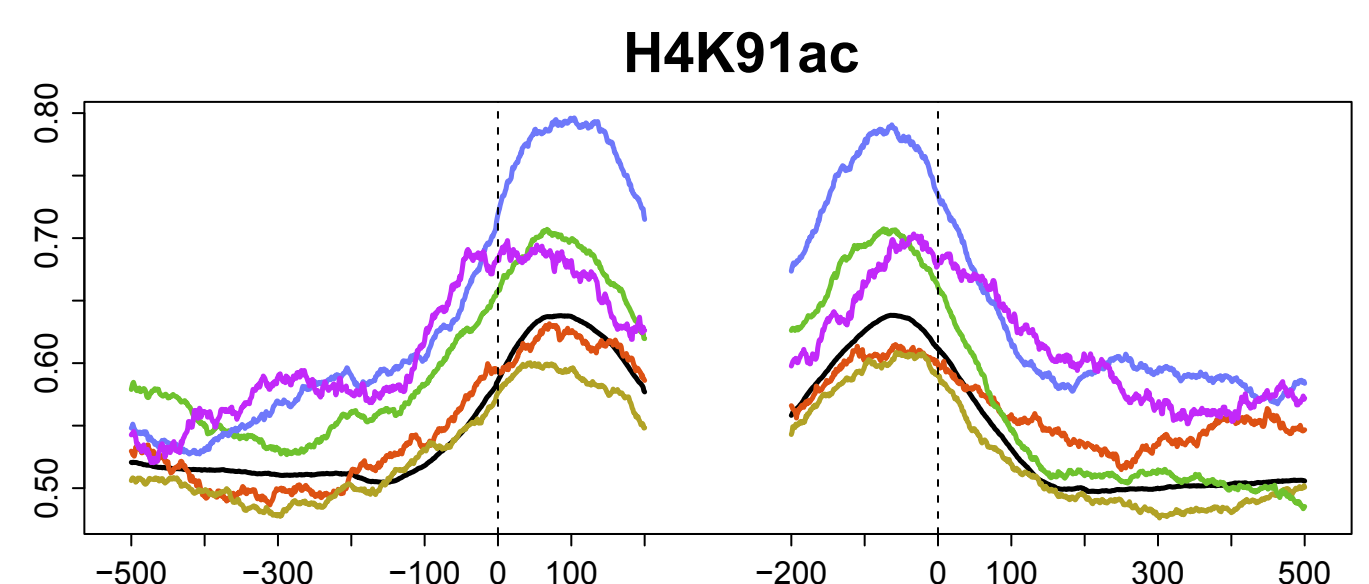

Intron Exon

Exon Intron

Intron Exon

Exon Intron

**Supplementary Figure-3 (Tian)**

Supplement: Additional file 5 — The association of additional histone acetylations with AS. This figure shows the distributions of histone acetylations not included in Figure 2. [file 1471-2164-13-123-S5.PDF]

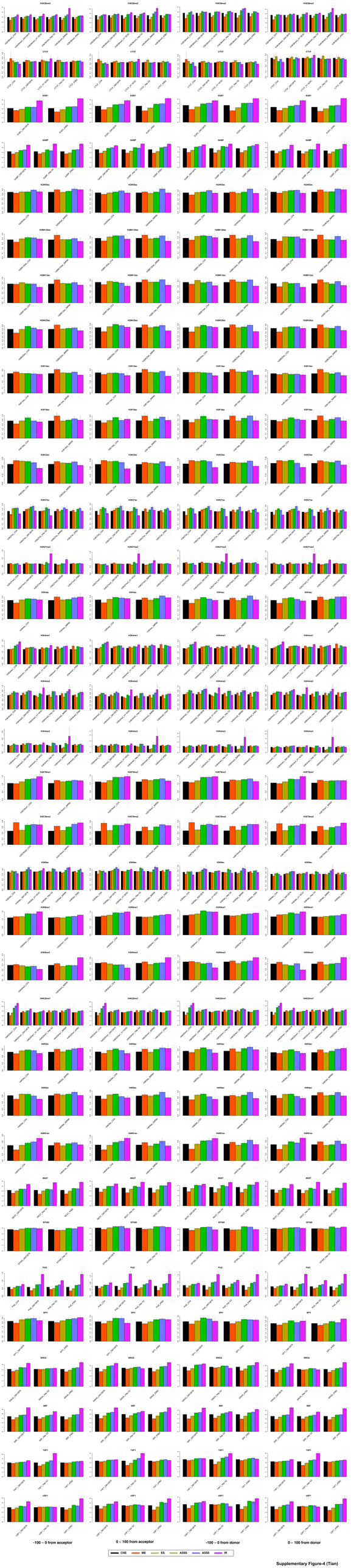

Supplement: Additional file 6 — The association of additional protein features with AS. This figure shows the profiles of protein features not included in Figure 3. [file 1471-2164-13-123-S6.PDF]

**A****EP300**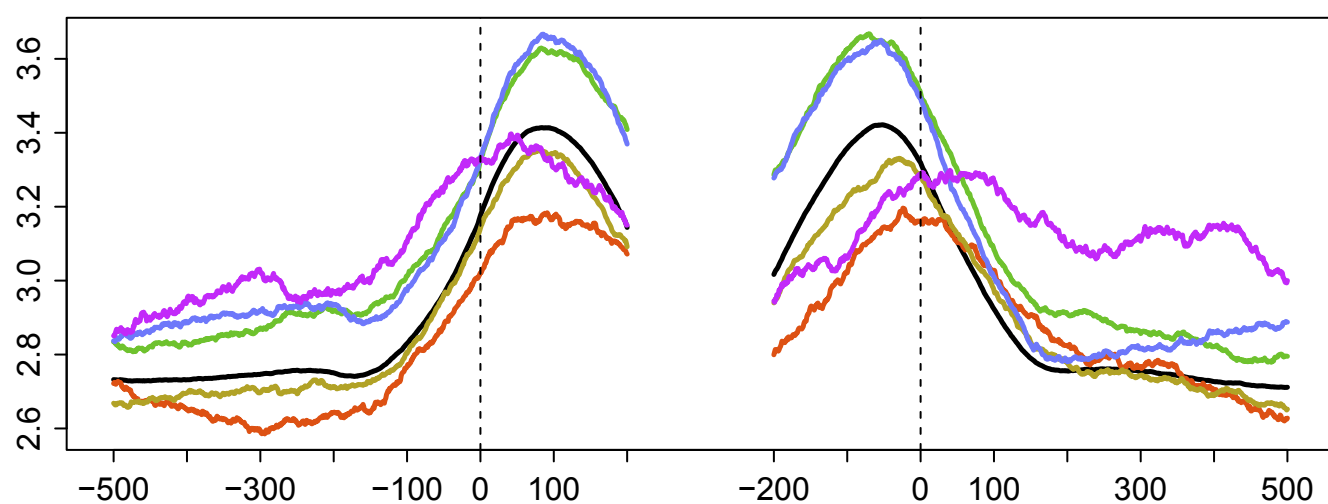**B****SPI1**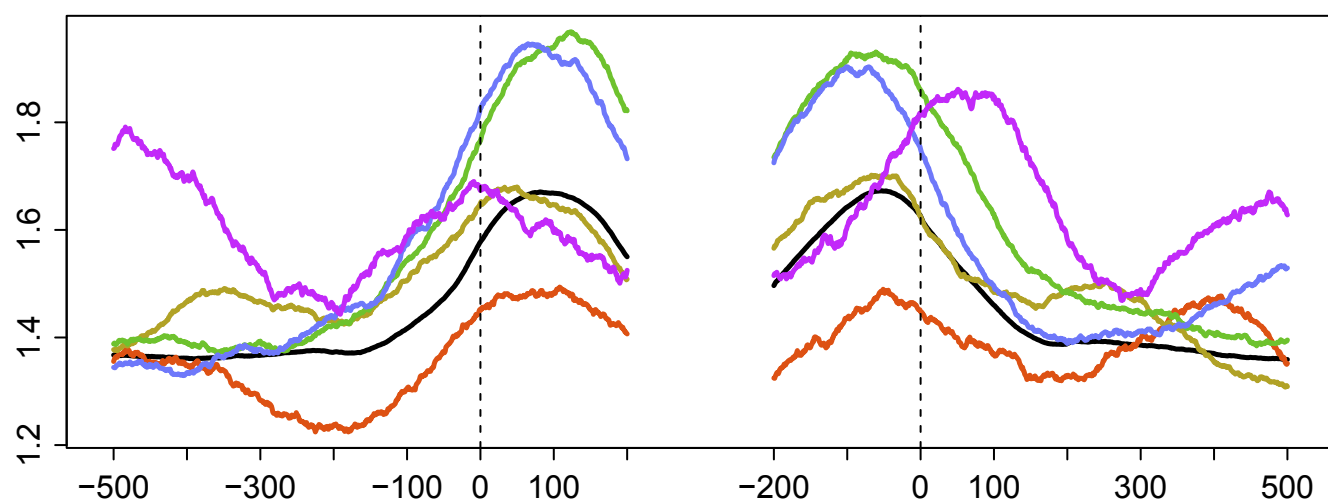**C****CTCF**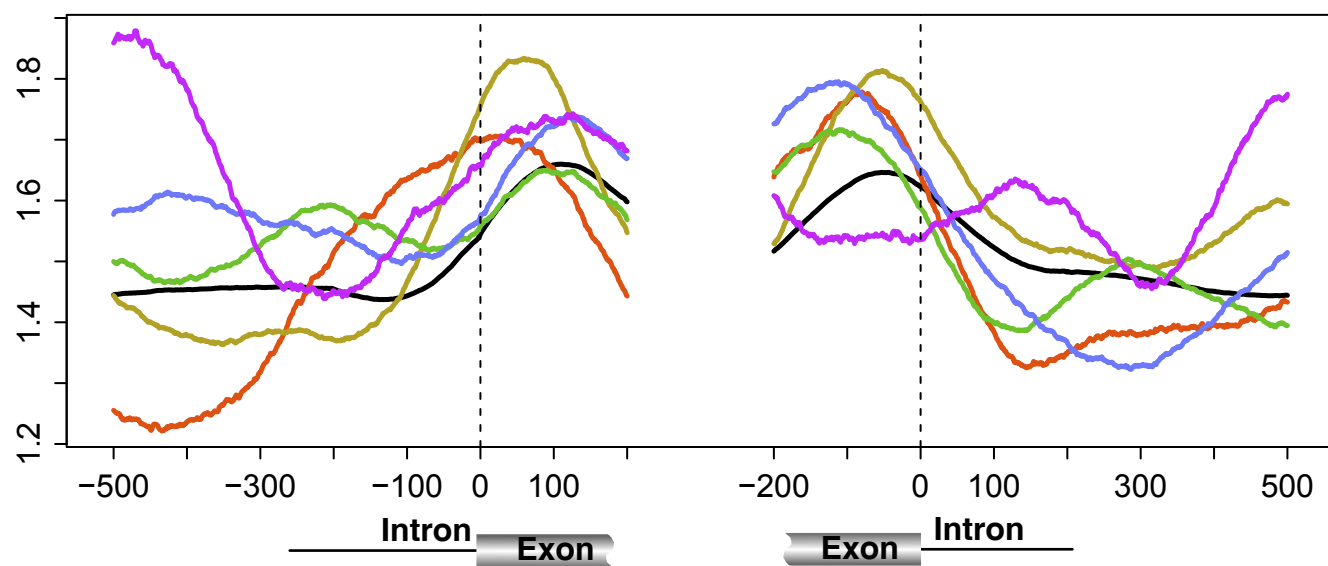

**CNE** **ME** **ES** **A3SS** **A5SS** **IR**

Supplement: Additional file 7 — The association of histone modifications and protein features with AS in different cell types. The level of each feature in four bins around the accepter and donor splice sites are shown for different cell lines; the error bars are drawn for 5% confidence interval. [file 1471-2164-13-123-S7.PDF]

# Classical Multidimensional Scaling

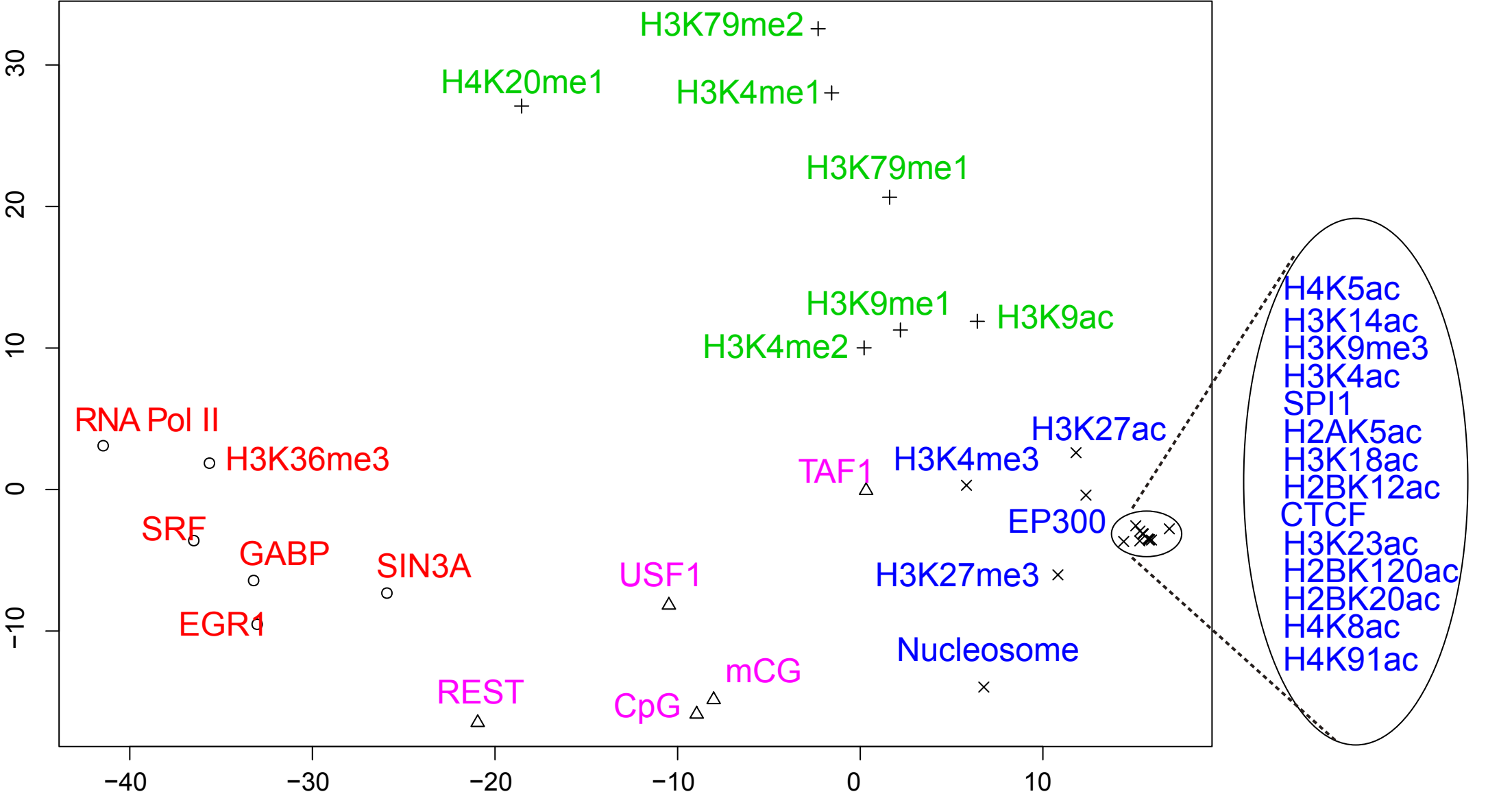

Supplementary Figure-6 (Tian)

Supplement: Additional file 8 — Classical multidimensional scaling plot of epigenetic features on the basis of their association with AS. The color and point type of each epigenetic features is the same with the cluster index of the k-means clustering result (Figure 4). The distance of two features indicates the closeness of their relationships. Note that cluster 1 and cluster 2 features are distantly to each other while cluster 3 and cluster 4 features are more closely related. Some features, such as nucleosome occupancy and NRSF, are located in between clusters. Classical multidimensional scaling is done using "cmdscale" function in GNU R. [file 1471-2164-13-123-S8.PDF]

Input\_IMR90

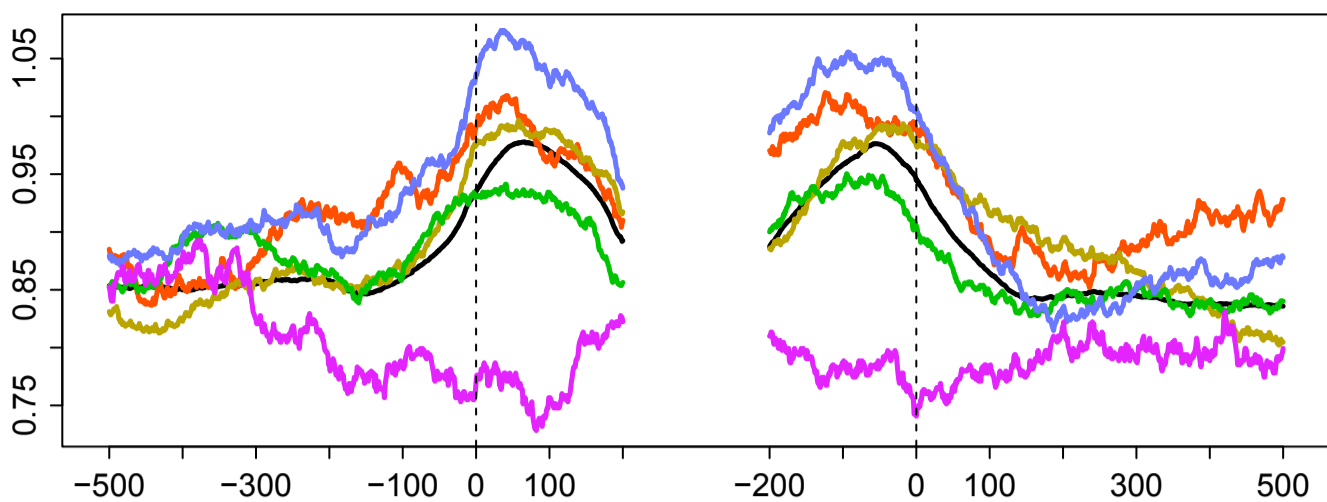

Input\_GM12878

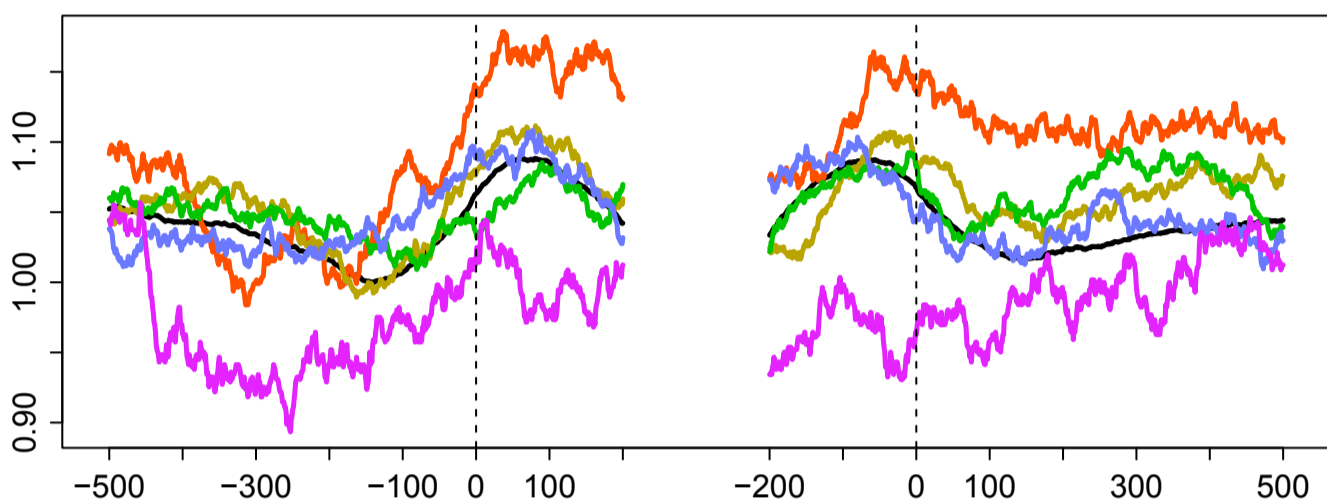

Input\_K562

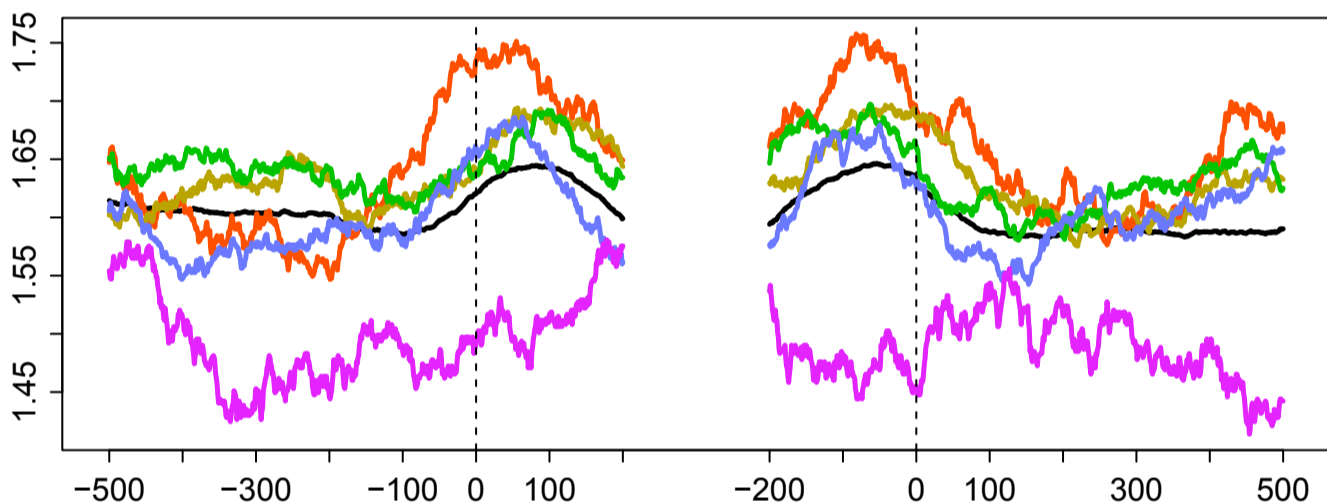

Input\_H1 hESC

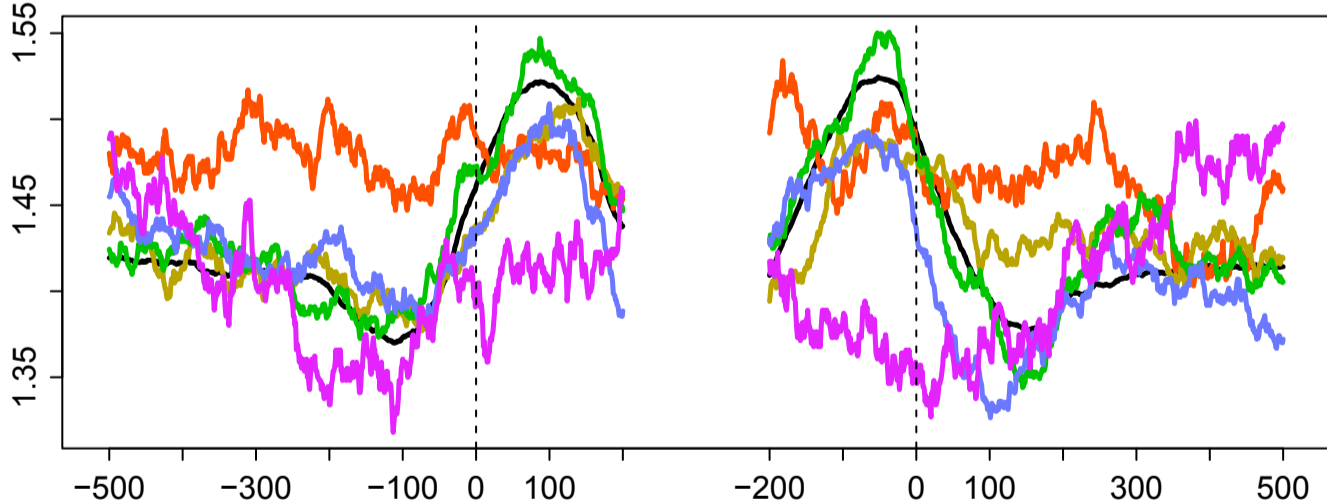

Input\_Hep G2

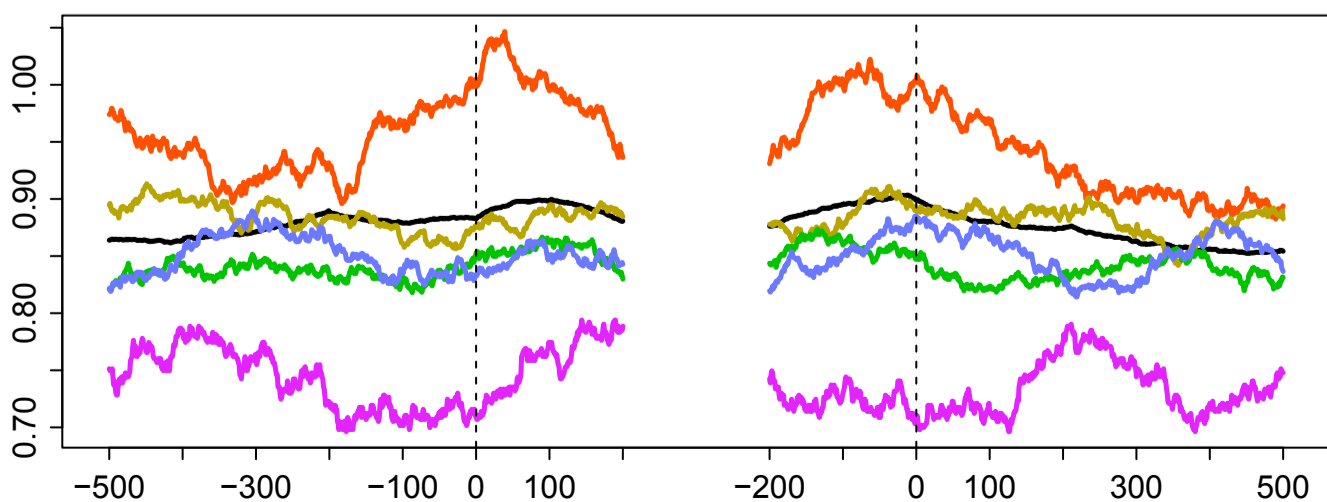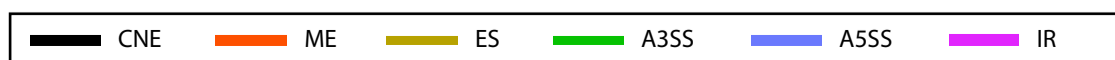

Supplement: Additional file 9 — The distribution of ChIP-seq input data in different types of ASEs and CNEs. [file 1471-2164-13-123-S9.PDF]

Corrected

Uncorrected

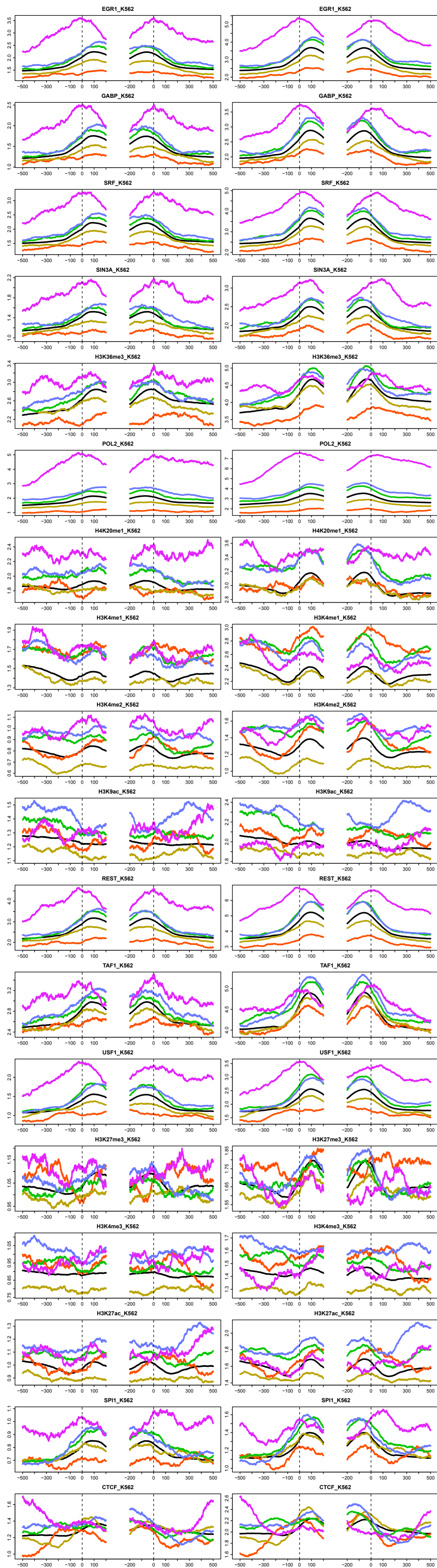

Corrected

Uncorrected

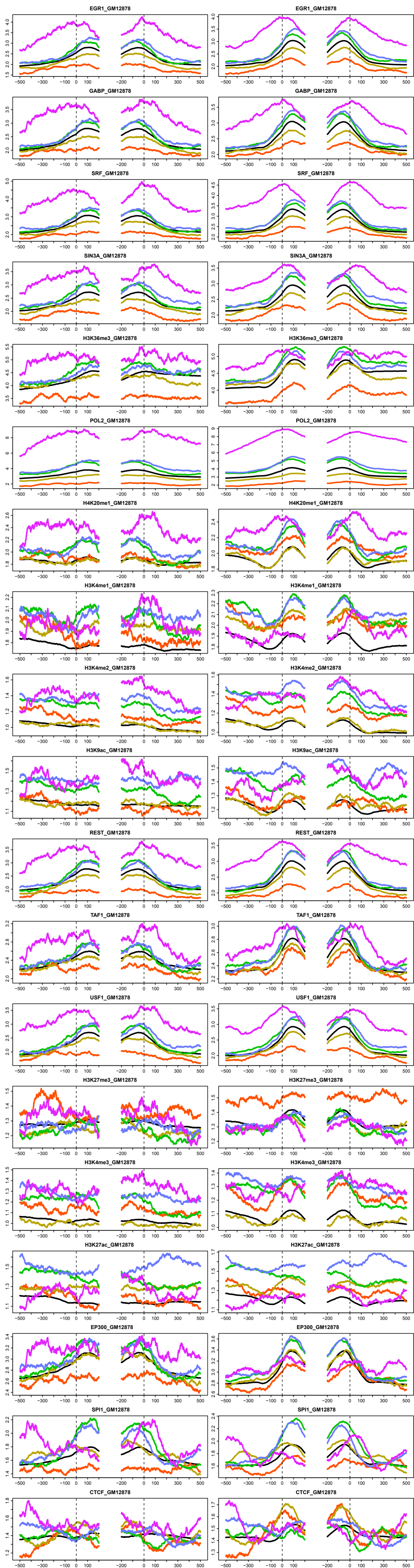

Corrected

Uncorrected

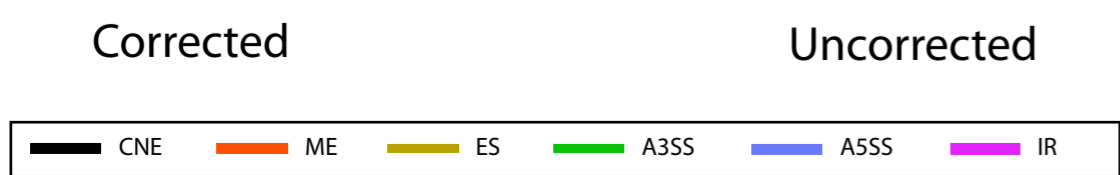

Supplement: Additional file 10 — The distribution of ChIP-seq data surrounding the splice sites of ASEs and CNEs in GM12878 and K562 cell lines. The patterns of most features are not changed after the correction. Only GM12878 and K562 cell lines are selected because they have available data for most of our significantly associated feature. [file 1471-2164-13-123-S10.PDF]

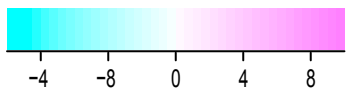

Corrected

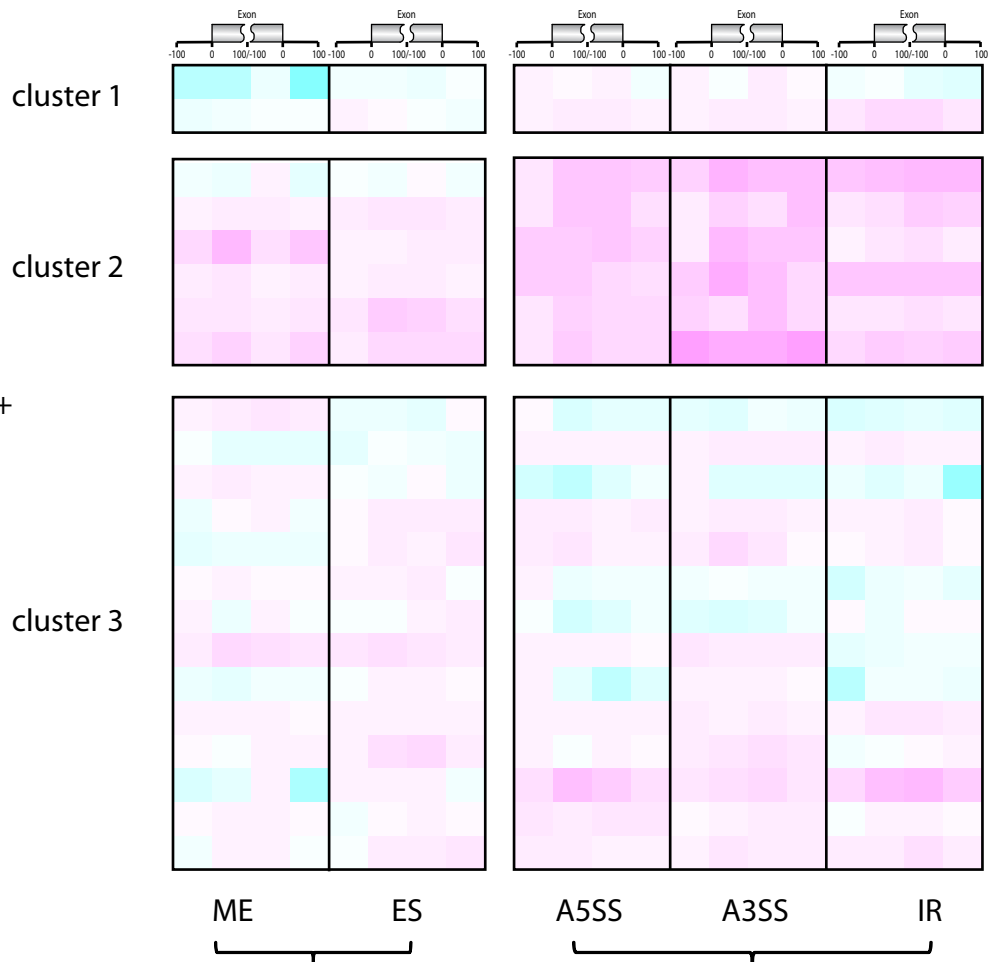

Uncorrected

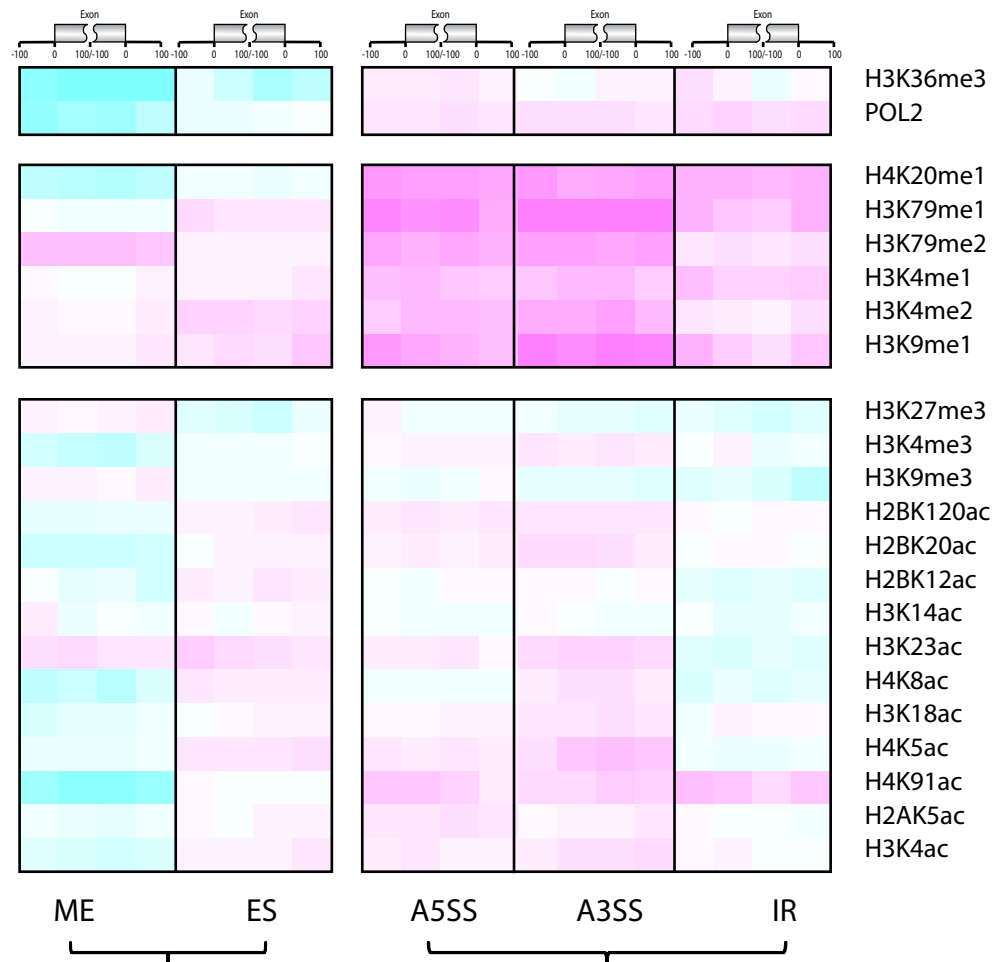

Supplement: Additional file 12 — Heatmap of epigenetic features corrected by nucleosome occupancy. Un-adjusted P-values are shown. The method is the same as that in Figure 4. [file 1471-2164-13-123-S12.PDF]

# CNE

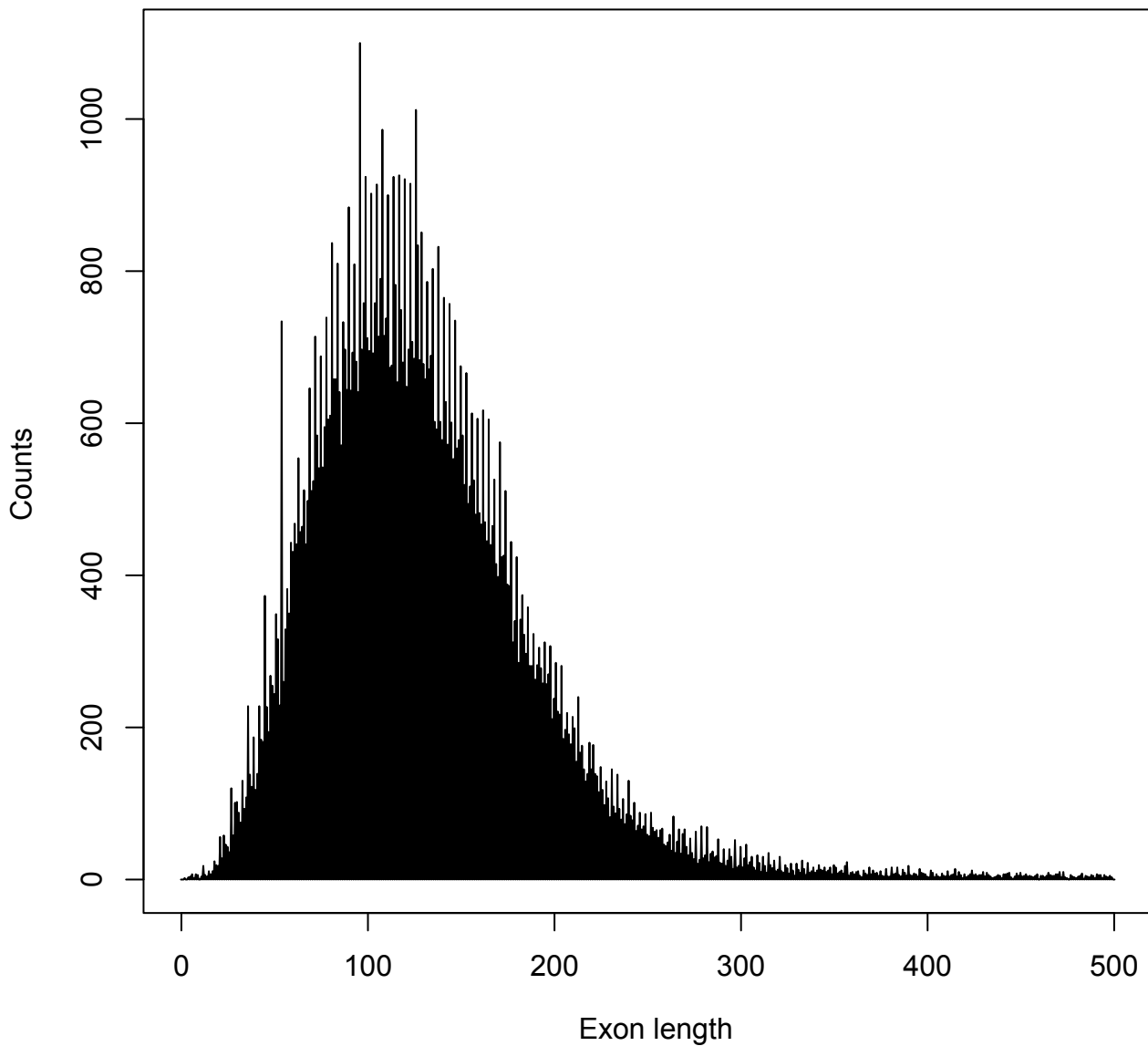

Supplement: Additional file 13 — The distribution of exon length. [file 1471-2164-13-123-S13.PDF]
