# Supplementary material for: Epigenetic features are significantly associated with alternative splicing
Source: BMC Genomics. 2012 Mar 29;13:123. doi: 10.1186/1471-2164-13-123 (PMC3362759; doi:10.1186/1471-2164-13-123)
Supplement: Additional file 11 — Heatmap of epigenetic features corrected by ChIP-seq input. Un-adjusted P-values are shown. The method is the same as that in Figure 4. [file 1471-2164-13-123-S11.PDF]

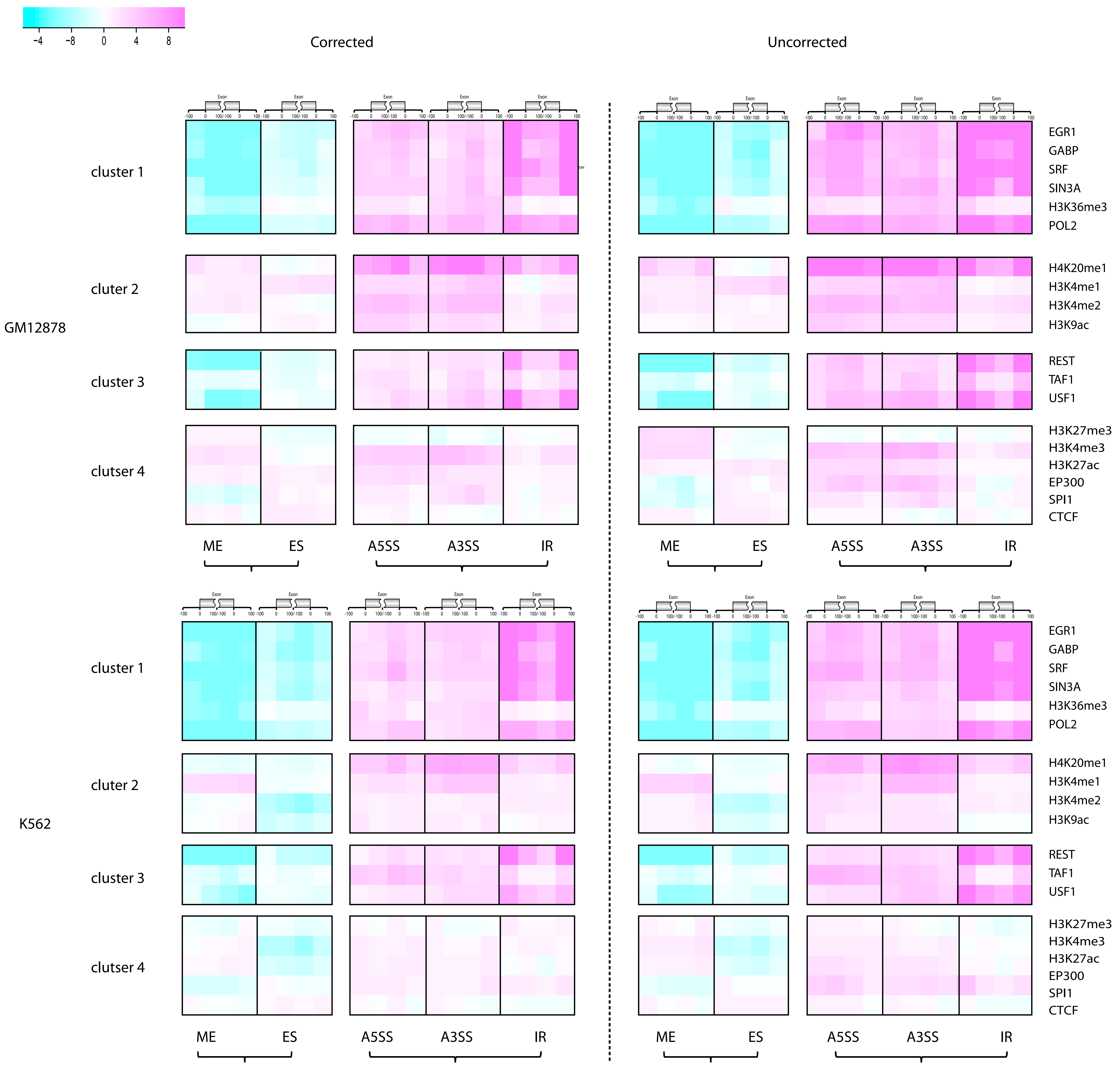

-4
-8
0
4
8
Corrected
Uncorrected
Exon
-100
0
100/-100
100
cluster 1
cluter 2
cluster 3
clutser 4
GM12878
K562
EGR1
GABP
SRF
SIN3A
H3K36me3
POL2
H4K20me1
H3K4me1
H3K4me2
H3K9ac
REST
TAF1
USF1
H3K27me3
H3K4me3
H3K27ac
EP300
SPI1
CTCF
ME
ES
A5SS
A3SS
IR
